# Electrospun PPDO-BG/PVDF fibrous membranes with piezoelectric synergistic bioactive ion controlled release for enhanced bone repair

**DOI:** 10.1093/rb/rbag120

**Published:** 2026-06-09

**Authors:** Qiuyu Zeng, Junqin Mao, Yutong Zhang, Xinyu Zhang, Tao Shen, Wei Tang, Heng Zheng, Guoyu Lv

**Affiliations:** College of Physics, Sichuan University, Chengdu 610065, China; College of Physics, Sichuan University, Chengdu 610065, China; College of Physics, Sichuan University, Chengdu 610065, China; College of Physics, Sichuan University, Chengdu 610065, China; College of Physics, Sichuan University, Chengdu 610065, China; College of Physics, Sichuan University, Chengdu 610065, China; College of Physics, Sichuan University, Chengdu 610065, China; College of Physics, Sichuan University, Chengdu 610065, China

**Keywords:** piezoelectric, bioactive glass, electrospinning, bone repair

## Abstract

Bone defects caused by trauma, infection or tumors remain challenging in clinics. Conventional methods cannot simultaneously regulate the electrical microenvironment, ionic microenvironment and extracellular matrix (ECM) microenvironment during osteogenesis. This study reports a piezoelectric and bioactive glass (BG)-loaded fibrous membrane (PVO) fabricated via electrospinning from polyvinylidene fluoride (PVDF) and poly(p-dioxanone) (PPDO). The PVDF component provides piezoelectric stimulation to build the electrical microenvironment, while BG-loaded PPDO enables sustained ion release to optimize the ionic microenvironment. The fibrous structure mimics the ECM microenvironment. *In vitro* osteogenic differentiation experiments using bone marrow mesenchymal stem cells (BMSCs) showed that the PVO fibrous membrane doped with 0.5% BG exhibited the highest alkaline phosphatase (ALP) content after 14 days of electrical stimulation combined with BG treatment, with substantial calcium nodules formed by day 21. *In vivo* implantation experiments in rat skull defect models further validated its therapeutic potential, with new bone formation observed in the PVO-0.5 group up to day 28. Its structure, piezoelectric performance and ion release synergistically improve osteogenic activity, making the PVO membrane an ideal scaffold for bone tissue engineering and providing an effective solution for bone repair.

## Graphical abstract

**Graphical Abstract:**
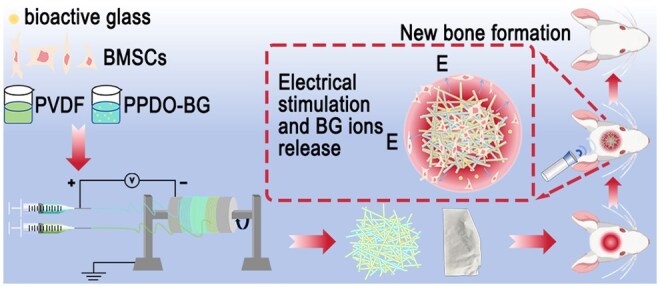

## Introduction

In recent decades, biomaterials have been widely developed for bone repair and regeneration, yet the repair of critical-sized bone defects remains a formidable clinical challenge [1–3]. Unlike small bone defects that can self-heal efficiently, critical-sized bone defects lack an appropriate supporting matrix, which greatly hinders osteoblast migration and spontaneous bone regeneration [4, 5].

Bone regeneration depends on the coordinated regulation of the local microenvironment, including the electrical microenvironment, ionic microenvironment, and extracellular matrix (ECM) microenvironment [6–10]. Traditional strategies such as autologous [11–13] and allogeneic bone grafts [14, 15], as well as conventional artificial bone substitutes [8, 16–18], are limited by donor scarcity, immune rejection, infection risk, and insufficient biological performance [8, 19]. Synthetic polymer scaffolds have emerged as promising candidates for bone tissue engineering due to their tunable mechanical properties, degradability and structural flexibility [6, 16, 17]. Therefore, there is an urgent need to develop novel biomimetic scaffolds that can simulate the aforementioned three microenvironments while exhibiting excellent biocompatibility, bone-inducing activity and appropriate mechanical properties.

Due to the representative piezoelectric properties of natural bone tissue, electroactive piezoelectric materials have garnered increasing attention [8, 20, 21]. Electroactive piezoelectric biomaterials can induce stem cell migration by establishing a local electrical microenvironment [20]. Piezoelectric polymer materials used to produce electrical stimuli include poly-L-lactic acid (PLLA), polyvinylidene fluoride (PVDF) and their copolymer poly(vinylidene fluoride-co-trifluoroethylene) (PVDF-TrFE) [8, 21]. Among these polymers, PVDF exhibits a relatively high piezoelectric coefficient. Additionally, PVDF has been widely applied in cardiac engineering [22, 23], neural regeneration [24, 25] and bone engineering [8, 21] due to its excellent biocompatibility and piezoelectric stability. Previous studies have confirmed that PVDF fibers can mimic the ECM structure and provide electrical stimulation to cells in a 3D microenvironment [26, 27]. This electrical stimulation activates the influx and activity of calcium ion channels, and the resulting calcium signals participate in signaling to promote bone formation [20, 28]. However, PVDF is a typical bioinert polymer with strong hydrophobicity and high brittleness, which greatly impairs cell adhesion and tissue integration, thus limiting its bone repair efficacy [29]. Therefore, it is necessary to introduce hydrophilic, flexible and biodegradable polymer materials and bioactive substances to overcome the limitations of PVDF. Poly(p-dioxanone) (PPDO) is a biodegradable aliphatic polyester with excellent biocompatibility, favorable mechanical flexibility, and controllable biodegradation behavior [30–32]. Electrospun PPDO fibrous membranes have been proven to possess good mechanical performance and sustained-release capacity [31]. Additionally, bone repair materials require sufficient ions, and bioactive glass (BG) has attracted significant attention in bone tissue engineering due to its outstanding osteoconductivity, osteoinductivity and bioactivity [10, 33–35]. The application of BG combined with biomedical polymers to fabricate tissue engineering scaffolds has been extensively reported in the field of bone tissue regeneration [10, 33]. During degradation, BG continuously releases silicon, calcium and phosphate ions, which may synergistically promote matrix deposition, biomineralization, osteoblast proliferation, and the expression of osteogenic genes and growth factors, thereby contributing to accelerated bone regeneration [10, 33–35].

Extensive studies have confirmed the independent roles of piezoelectric materials and bioactive ions in bone repair, and the integration of piezoelectricity with controllable ion release has become a rapidly developing frontier [36–38]. However, current composite systems have critical limitations. Dong et al. [38] developed PVDF-coated PCL-TCP scaffolds with pulsed electromagnetic field (PEMF) stimulation, which mainly relied on external PEMF and may not generate self-sustained electrical signals *in vivo*, with weak interfacial adhesion and limited structural stability. TiO_2_@PVDF piezoelectric nanofiber membranes can regulate osteogenic differentiation but lack bioactive components for sustained ion release, failing to form a stable ionic microenvironment [37]. Zhao et al. [36] fabricated PVDF/BG scaffolds via simple physical stacking to mimic the periosteum. This design has several flaws: it exhibits weak interfacial bonding, leading to a tendency for delamination that may reduce piezoelectric performance. It also has poorly controlled BG ion release, which hinders the synergistic effect between piezoelectric stimulation and ionic regulation.

To address these bottlenecks, this study adopted an alternating electrospinning strategy and utilized PPDO as a dedicated carrier for BG to construct PPDO-BG/PVDF (PVO-X) composite fibrous membranes. This design targeted and remedied three core deficiencies in bone repair: piezoelectric regulation derived from PVDF endowed the scaffold with stable electrical signal output, helping to reconstruct the electrical microenvironment required for osteogenesis and initiating early cell response and osteogenic differentiation. Controllable ion release enabled by the PPDO carrier achieves relatively uniform and sustained BG ion delivery, reducing burst release and helping to optimize the ionic microenvironment to support subsequent mineralization and bone maturation. Biomimetic ECM structure constructed by electrospinning provides favorable cell adhesion and proliferation sites, simulating the natural ECM microenvironment of bone tissue.

Collectively, the integration of PVDF piezoelectricity, PPDO-mediated BG sustained release and biomimetic fibrous structure helped to simultaneously optimize the electrical microenvironment, ionic microenvironment, and ECM microenvironment, contributing to efficient synergistic osteogenesis.

In this work, the PVO-X fibrous membranes were fabricated via alternating electrospinning of PVDF and BG-doped PPDO. First, we characterized the physicochemical properties of the scaffold, including surface morphology, mechanical properties, piezoelectric performance, and hydrophilicity. Subsequently, we investigated the synergistic osteogenic effects of piezoelectric stimulation and BG ion release through *in vitro* degradation, mineralization experiments, and bone marrow mesenchymal stem cell osteogenic differentiation models. Finally, the *in vivo* bone repair potential of PVO-X fiber membranes was evaluated using a rat skull critical-sized bone defect implantation model. Through multidimensional *in vitro* and *in vivo* experiments, this study systematically demonstrated the feasibility and potential of the PVO-X fibrous membranes in bone repair applications. It aimed to provide a novel solution with both bioactive and physicochemical modulation functions for bone defect repair and to offer experimental evidence and theoretical references for future research.

## Experimental sections

### Materials and methods

Poly(p-dioxanone) (PPDO, Degussa GmbH, Germany); polyvinylidene fluoride (PVDF, M_w: 1 000 000, Arkema, France); BG (45S5) was provided by East China University of Science and Technology. Hexafluoroisopropanol (HFIP) ((CF_3_)_2_CHOH ≥ 99.0%, Shanghai Aladdin Biochemical Technology Co., Ltd); N, N-dimethylformamide (DMF) and acetone (Kelon Chemical Company, Chengdu, China).

The PVO-X nanofiber scaffolds were prepared through alternating electrospinning of PVDF and PPDO. 1.6 g PVDF was dissolved in 20 mL of DMF/acetone solution at 30°C and stirred overnight. Additionally, different mass fractions of BG powder were added to 20 mL of HFIP and thoroughly mixed by ultrasonic homogenization for 1 h, with BG concentrations of 0%, 0.1%, 0.5% and 1%. The resulting BG solutions were then mixed with 1.6 g PPDO and stirred at room temperature for 4 h in a sealed, light-proof container to obtain homogeneous PPDO-BG spinning solutions. The PPDO-BG and PVDF spinning solutions were transferred to spinning syringes and spun under the following conditions: 22 kV voltage, 1 mL/h flow rate, 200 rpm roller speed, 10 mm/s moving device speed, and 15 cm electrospinning distance. The resulting fiber membranes were named PVO-0, PVO-0.1, PVO-0.5 and PVO-1 based on the BG concentration.

### Scanning electron microscopy

Scanning electron microscopy (SEM) was used to analyze the morphology and microstructure of PVO fiber membranes. Energy dispersive spectroscopy (EDS) was employed to investigate the elemental distribution on the sample surfaces. The fiber diameter of each sample was measured from SEM images using ImageJ software.

### Fourier transform infrared spectroscopy analysis

The Fourier transform infrared spectroscopy (FTIR) analysis was performed on the PVDF powder raw material, PVDF fiber membrane, PPDO fiber membrane, and a series of PVO-X fiber membranes using the Nicolet-560 Fourier Transform Infrared Spectrometer from the United States.

### X-ray diffraction

The X-ray diffraction (XRD) analysis of PVDF fiber membrane, PPDO fiber membrane, and a series of PVO fiber membranes was performed under the following conditions: working voltage of 40 kV, current of 25 mA and scanning rate of 0.2 degrees/min.

### Piezoelectric experiment

The PVDF and PVO-X films were cut into 30 mm × 20 mm rectangular pieces, with conductive copper tapes attached to both sides, and then impacted with a force of 1 Hz and 5 N.

### Water contact angle

The water contact angle of the membrane was measured by optical contact angle meter (KRUSS DSA25, Germany).

### Mechanical properties

The tensile properties of PVO-X fiber membrane samples were evaluated using an electronic universal testing machine (INSTRON, USA). The samples had a width of 10 mm, length of 60 mm and thickness of 0.06 mm. The testing machine stretched the PVO-X fiber membrane at a rate of 100 mm/min until fracture, thereby assessing the ultimate elongation strain and maximum tensile strength of the samples.

### Hemocompatibility assay

Using rabbit blood with 3.2% sodium citrate as anticoagulant, erythrocytes were isolated by centrifugation at 1200 rpm for 10 min. The obtained erythrocytes were washed three times with PBS and diluted to a 5% concentration. Subsequently, a 6 cm^2^ membrane was mixed with 1 mL of erythrocyte suspension, incubated at 37°C for 1 h under shaking, and centrifuged at 1200 rpm for 5 min. Then, 100 μL of the supernatant was transferred to a 96-well plate, and the absorbance of the supernatant was measured at 540 nm using a Multiskan ELISA reader (A_sample_). The absorbance of erythrocytes treated with 0.1% Triton X-100 and PBS served as the positive control (A_triton_) and negative control (A_PBS_), respectively. The hemolysis rate of the samples was calculated using the following formula:


$$\text{Hemolysis}\;\text{ratio}\;(\%)=\frac{A_{\text{sample}}-A_{\text{PBS}}}{A_{\text{triton}}-A_{\text{PBS}}}\;\times 100\%.$$

### Cytocompatibility assay

Bone marrow mesenchymal stem cells (BMSCs) were used for *in vitro* experiments. The cells were cultured in an incubator (37°C, 5% CO_2_) until the cell count and morphology were healthy. A 60 cm^2^ sample was thoroughly mixed with 10 mL of cell culture medium to ensure complete immersion. After 24 h, the mixture was centrifuged at 1200 rpm for 3 min to obtain the sample extract. BMSCs were seeded in a 96-well plate, and after cell adhesion, the medium was replaced with the sample extract. Cell viability was assessed on days 1, 3 and 5 using the Cell Count Kit-8 (CCK-8, UElandy, C6005M, China). Additionally, a live/dead dyeing kit containing calcein-AM (green fluorescence) and ethidium-1 (red fluorescence) (UElandy, L6023S, China) was used to evaluate the viability of BMSCs. The morphology of BMSCs was detected using phalloidin and 4′,6-diamidino-2-phenylindole (DAPI).

### Cell migration assay

The BMSCs were seeded at a density of 2 × 10^5^ cells per well in a 24-well plate and cultured until a dense monolayer formed. A 100 μL vertical scratch was created using a pipette tip, followed by gentle rinsing with PBS to remove detached cells. Serum-free extract solutions from each treatment group (material groups) and standard serum-free culture medium (blank control group) were added accordingly. Images were captured under an inverted microscope at 0 h and 24 h, and the scratch area was measured using ImageJ software. The scratch healing rate was calculated using the following formula:


$$\text{Scratch}\;\text{Healing}\;\text{Rate}\;(\%)\;=\frac{W_{0}-W_{t}}{W_{0}}\times 100\%,$$

where *W*_0_ represents the initial average scratch area at 0 h and *W*_t_ represents the average scratch area after 24 h of culture.

### *In vitro* degradation experiments

The experiment was conducted at a concentration of 6 cm^2^/mL, with initial mass recorded as *W*_0_. The samples were placed in a 37°C shaker for degradation, with PBS replaced every three days. Three parallel samples were prepared for each scaffold group. After 1, 4, 7, 14, 28 and 56 days of degradation, the fibrous membranes were dried and their post-degradation mass recorded as *W*_1_. The mass loss rates of different fibrous membranes at various degradation stages were calculated using the provided formula.


$$\text{Weight}\;\text{loss}\;(\%)\;=\frac{W_{0}-W_{1}}{W_{0}}\times 100\%.$$

The surface morphology of the degraded scaffold was analyzed using SEM and EDS. The degradation solution of the scaffold was tested with a pH meter. The ion release behavior of the scaffold was evaluated by measuring the concentration of silicon ions in the degradation solution using inductively coupled plasma atomic emission spectrometry (ICP-AES, Varian 720).

### *In vitro* mineralization experiment

The mineralization solution of simulated body fluid (SBF, pH = 7.4) was prepared at a concentration of 6 cm^2^/mL and incubated in a 37°C shaker for *in vitro* mineralization. Three parallel samples were set up for each group. The fibrous membranes after mineralization at 1, 4, 7, 14 and 28 days were analyzed for surface morphology of the degraded scaffolds using SEM and EDS.

### *In vitro* osteogenic differentiation experiments

The prepared square fibrous membranes (side length 15 mm) were placed in 24-well plates and fixed with polystyrene rings of the same material as the cell culture plates, followed by sterilization. To evaluate the differences in osteogenic differentiation ability of PPDO, PVDF, PVO-0 and PVO-0.5, BMSCs were seeded on the sterilized samples. After 2 days of culture, the complete medium was replaced with osteogenic induction medium and continued to be cultured, with the osteogenic induction medium being changed every 48 h. Subsequently, ultrasonic stimulation was applied daily. The alkaline phosphatase (ALP) activity of BMSCs on the fibrous membranes on days 7 and 14 were qualitatively and quantitatively detected using the BCIP/NBT alkaline phosphatase chromogenic kit and the ALP kit. The calcified nodules on the fibrous membranes on days 14 and 21 were qualitatively and quantitatively assessed using Alizarin Red S (ARS) staining and dodecylpyridine. Additionally, the morphology of the calcified nodules was observed using SEM.

### Statistical analysis

All quantitative results were expressed as mean ± standard deviation. The independent samples t-test was used for pairwise comparisons. A *P* < 0.05 was considered statistically significant. The *in vivo* experiment had a relatively limited sample size (three effective rats per group per time point), which may affect the statistical robustness. To minimize this impact, we used SPSS software for rigorous statistical analysis (including homogeneity of variance test and rank sum test when variance is uneven) to ensure the reliability of the results. The limitations of the sample size are discussed in detail in the discussion section, and we will expand the sample size in subsequent studies to further verify the repeatability of the results.

### Animal experiments

A total of 18 male rats were selected for *in vivo* studies. After a 7-day acclimation period, the rats were randomly assigned to one of three groups: control group, PVO-0.5 group and PVO-0 group. Samples were collected at 4 weeks and 8 weeks postoperatively for further histological and biochemical analysis.

#### Model establishment

All rats were prepared for craniotomy defect models. Preoperative intramuscular antibiotic injection was administered to prevent infection. A midline longitudinal incision was made in the scalp, and a 5 mm diameter critical defect was created in the unilateral temporal region using a craniotomy drill.

#### Material implantation

PVO-0.5 and PVO-0 fibrous membranes were overlapped the defect edge by approximately 0.3 cm, and the edges were fixed with medical-grade biological adhesive. The control group received no intervention. The incision was sutured with 3-0 silk thread, and intramuscular antibiotics were administered once daily for 1–3 days postoperatively to prevent infection.

#### Ultrasonic-induced piezoelectric stimulation

Ultrasonic stimulation was performed daily in each group 24 h after operation, and three animals were selected from each group for relevant detection at 4 weeks and 8 weeks after implantation.

#### HE and Masson staining

At 4 weeks and 8 weeks post-modeling, the tissue was fixed in 10% neutral formaldehyde, decalcified with EDTA, routinely embedded, sectioned and stained for histological analysis. The fixed tissues were stained with HE and Masson’s stain at 4 weeks and 8 weeks after modeling. Each section was first observed under 20× magnification to examine the entire tissue, followed by image acquisition under 400× magnification based on tissue size and expression patterns. Collagen fibers (arrangement, orientation, quantity) and bone tissue distribution were then observed.

Ethical approval was obtained from the Ethics Committee of Sichuan Lilaisinuo Biotechnology Co., Ltd. (Certification No.: LLSN-2025171).

## Results and discussion

### Physical and chemical properties of PVO-X fiber membrane

**Figure 1:**
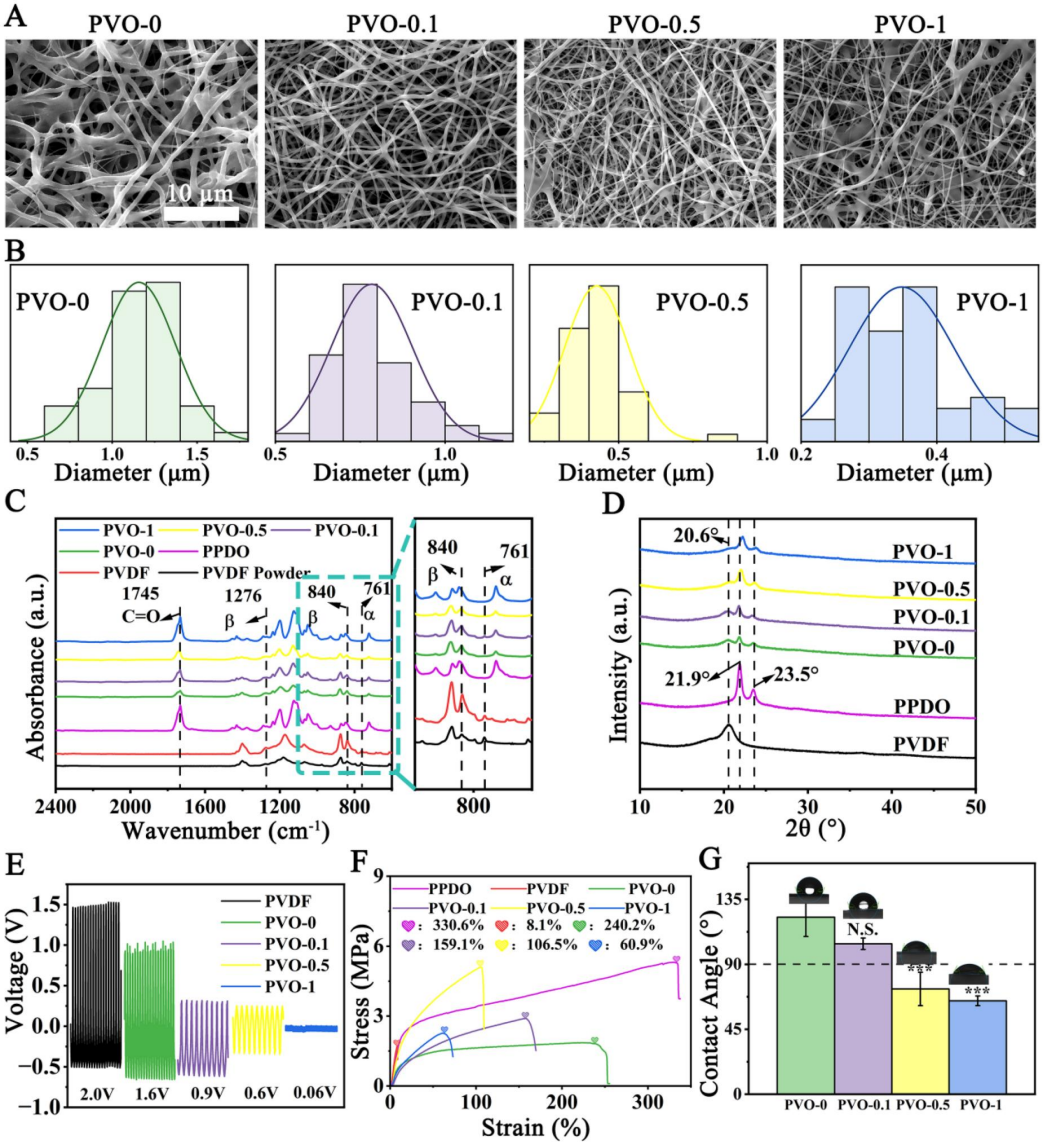
Physicochemical properties of PVO-X fiber membranes. (**A**) SEM images of fiber membranes; (**B**) Fiber diameter distribution; (**C**) Infrared spectra; (**D**) XRD patterns; (**E**) Piezoelectric output; (**F**) Stress-strain curves; (**G**) Water contact angle.

The morphology of the composite fiber membranes PVO-0, PVO-0.1, PVO-0.5 and PVO-1 were observed by SEM. As shown in Figure 1A, SEM images revealed uniform fiber dimensions, relatively smooth surfaces, and a highly porous network structure. Fiber diameter distribution analysis using ImageJ (Figure 1B) demonstrated that the addition of BG resulted in more uniform fibers and progressively smaller average diameters in PVO-0.1, PVO-0.5 and PVO-1 composite fiber membranes compared to the PVO-0 group. As shown in Supplementary Figure S2, the diameter decreased from 1.16 μm in the PVO-0 group to 0.35 μm in the PVO-1 group, forming macropores. Such randomly arranged fiber membranes could mimic the extracellular matrix and provided more adhesion sites for cells, facilitating cell adhesion and growth [26]. Furthermore, EDS (Supplementary Figure S3) revealed a uniform distribution of BG. As the BG content increased, the Si signal intensity also rose, indicating that the incorporation of BG into the PPDO matrix was consistent with experimental expectations.

The infrared spectrum (Figure 1C) revealed that the stretching vibration absorption peak of PPDO’s ester carbonyl group (C = O) appeared at 1745 cm^−1^, while the C-O bond stretching vibration absorption peaks in the 1200–1400 cm^−1^ range were characteristic of ether/ester bonds in PPDO molecules [31]. Measurements of PVDF powder and electrospun fiber membranes showed that the 840 cm^−1^ and 1276 cm^−1^ peaks in the infrared spectrum corresponded to the β-phase characteristic peaks of PVDF, while the 761 cm^−1^ peak represented the α-phase characteristic peak [26, 27]. The higher intensity of α-phase peaks compared to β-phase peaks in PVDF powder indicated that the primary crystal form was the non-polar α-phase. The β-phase characteristic peaks (840 cm^−1^ and 1276 cm^−1^) in PVDF showed significant enhancement, whereas the α-phase characteristic peak (761 cm^−1^) exhibited marked weakening or even disappearance, demonstrating that high-voltage electrospinning and stretching effectively promoted the crystalline transformation of PVDF from the non-polar α-phase to the polar β-phase. In the PVO composite system, both PPDO and PVDF characteristic peaks were observed, confirming the successful preparation of the composite fiber membrane. The addition of PPDO and a small amount of BG did not reduce the intensity of the β-phase peaks, but the infrared images of PVO-1 with 1% BG showed virtually no β-phase peaks, resembling those of pure PPDO. The β-phase was the key crystal form enabling PVDF to achieve piezoelectric functionality [26], which allowed the fiber membrane to exhibit higher piezoelectric output and provided a structural foundation for subsequent functional applications of the material.

As shown in Figure 1D, pure PPDO exhibited distinct crystalline peaks at 21.9° and 23.5°, corresponding to its typical crystal structure [30, 31]. The PVDF fiber membrane displayed a characteristic diffraction peak at 20.6°, consistent with its β-crystal form, while no significant α-crystal characteristic diffraction peaks were observed, which aligned with the infrared results [26, 27]. The XRD curves of the PVO hybrid fiber membrane showed diffraction signals at 20.6°, 21.9° and 23.5°, indicating that both PPDO and PVDF retained their respective crystalline characteristics after blending, albeit with reduced peak intensity, suggesting that the blending process may have partially diminished crystallinity. 45S5 BG is a typical amorphous bioglass. The addition of BG introduced a substantial amorphous phase into the system, reducing the proportion of polymer crystalline phases per unit volume [39]. The crystal structure of the PVO-X composite membrane showed minimal changes, remaining largely consistent with that of the PVO fiber membrane. Notably, the PVO-1 group exhibited almost no characteristic diffraction peak at 20.6°, which corresponded to the infrared results.

As shown in Figure 1E, the PVDF fiber membrane after electrospinning exhibited excellent piezoelectric properties, generating 2 V voltage under 5 N force. The addition of PPDO reduced the PVDF content, resulting in a slight decrease in PVO’s piezoelectric performance. Notably, the incorporation of BG significantly impacted the membrane’s piezoelectric performance, with a marked reduction in output voltage. However, a 0.5 V output was still achievable. Literature indicated that a voltage around 0.5 V could significantly promote osteogenic differentiation in cells, demonstrating that the voltage generated by the PVO-0.5 fiber membrane was sufficient for functional applications [40].

Figure 1F presented the stress–strain curves of each fiber membrane. Comparative analysis revealed that the pure PPDO fiber membrane exhibited a tensile strain of 330.6%, while the pure PVDF fiber membrane demonstrated brittleness, forming a striking contrast with the PPDO membrane. The composite fiber membrane combining both materials showed a maximum tensile strain reduction to 240.2% and a significant decrease in maximum stress. As the BG concentration increased, the maximum tensile breaking strength progressively decreased, with the maximum stress initially increasing and then decreasing. At a 0.5% BG concentration, the maximum tensile stress reached 5.1 MPa, and the maximum tensile breaking strength remained at 106.5%. Water contact angle measurements (Figure 1G) showed that the PVO-X fiber membrane transitioned from hydrophobic to hydrophilic with increasing BG concentration, which facilitated cell adhesion and growth.

### *In vitro* cellular compatibility, hemocompatibility

**Figure 2:**
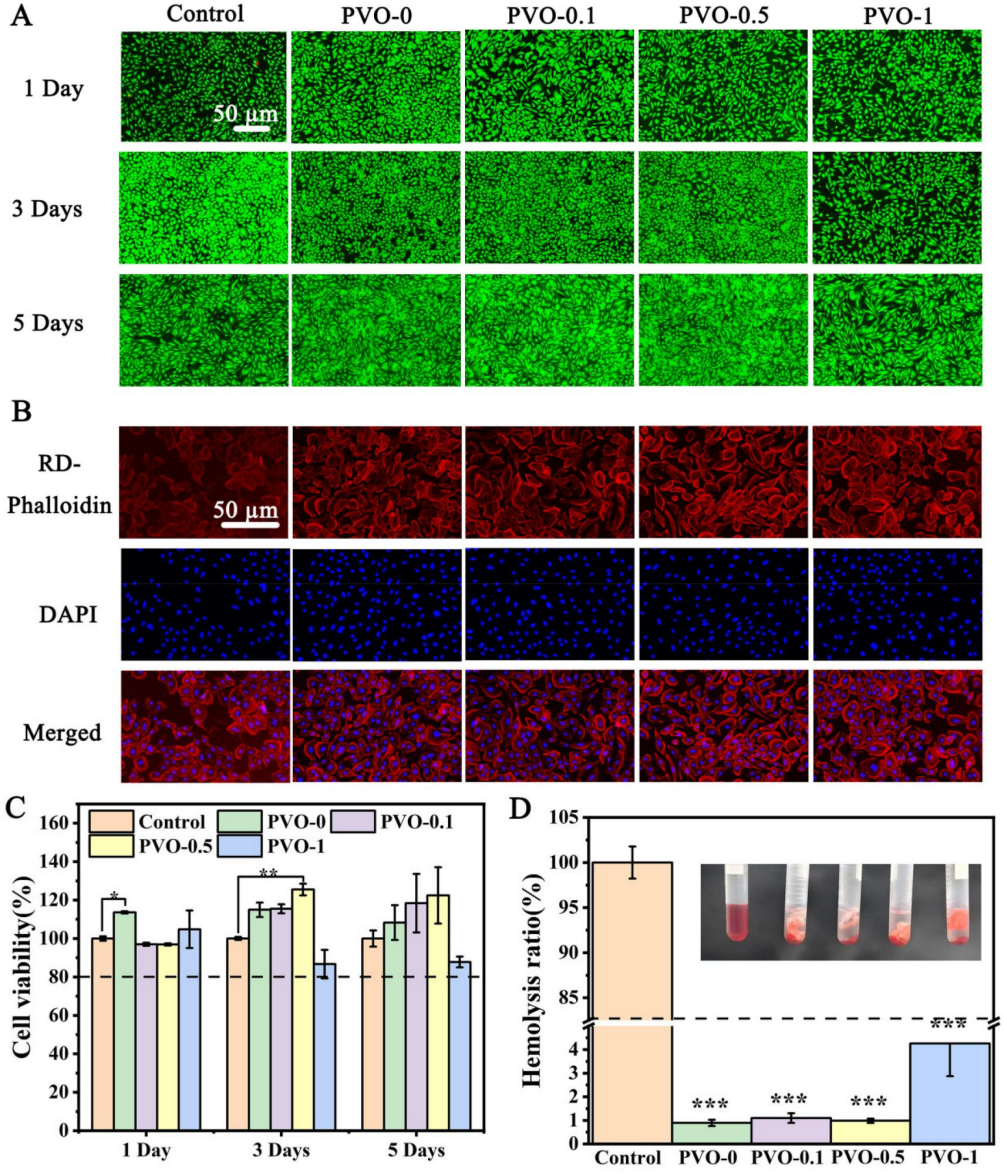
Cellular experiments and blood compatibility of PVO-X fibrous membranes. (**A**) Live/dead staining of cells cultured on PVO-X fibrous membranes; (**B**) Cytoskeleton staining images; (**C**) CCK-8 assay results; (**D**) Hemolysis test results. (*P < 0.05, **P < 0.01, ***P < 0.001, N.S., *P* > 0.05).

As shown in Figure 2C, the proliferation of BMSCs was evaluated on 1 day, 3 days and 5 days in each group. The results demonstrated that, compared to the control group, the PVO-0.1 and PVO-0.5 groups exhibited higher cell viability. Specifically, the PVO-0.5 group achieved a proliferation rate exceeding 120% on both day 3 and day 5. In contrast, the PVO-1 group showed a decline in proliferation rate to below 100%, although it still maintained a proliferation rate above 80%. These findings indicated that all fibrous membrane groups exhibited excellent biocompatibility, with the PVO-0.5 group demonstrating a significant capacity to promote cell proliferation. This cell-promoting effect and cytotoxicity were likely attributed to the degradation of BG, which releases ionic byproducts such as silicon, calcium, and phosphate into the surrounding culture medium. These ions are biocompatible or even beneficial at low concentrations, but excessive concentrations can disrupt cellular homeostasis, leading to cytotoxicity and cell death [31, 34]. The hemolysis test (Figure 2D) revealed that all concentrations of the fibrous membrane exhibited a hemolysis rate below 5%, indicating excellent biocompatibility. Notably, the hemolysis rate of the PVO-1 group approached 5%, consistent with the CCK-8 assay results.

Cell viability was further evaluated through live/dead staining to confirm cytotoxicity. As shown in Figure 2A, the cell density increased over time in all groups. Throughout the culture period, the cell density of PVO-0.5 was significantly higher than that of the control group on days 3 and 5, indicating enhanced cell proliferation. However, PVO-1 exhibited lower cell density and fewer viable cells compared to the control group, with these live/dead staining results consistent with the CCK-8 assay findings.

The cell morphology was further evaluated using DAPI and rhodamine phalloidin staining to assess cytocompatibility. As shown in Figure 2B and Supplementary Figure S6, all groups maintained morphological consistency with the control group, with clear cytoplasmic and nuclear structures after staining, which was consistent with the results of CCK-8 and live/dead staining assays. These experiments collectively demonstrated that PVO-X exhibited excellent biocompatibility. In subsequent experiments, we will further investigate its biological activity through *in vitro* mineralization.

The results of the cell migration experiments are shown in Supplementary Figures S7 and S8. At 24 h, the scratch healing rate in the PVO-0.5 group exceeded 90%, with the scratches nearly completely closed, demonstrating a significantly superior migration-promoting effect compared to other groups. The healing rates in the PVO-0, PVO-0.1 and PVO-1 groups were comparable, showing no clear advantage; all experimental groups exhibited better outcomes than the control group. These results were highly consistent with the findings of the CCK-8 cell proliferation assay and the ion release data. Appropriate concentrations of Si^4+^ ions significantly upregulate the expression of migration-promoting factors such as VEGF and bFGF, activating cell migration-related signaling pathways and thereby accelerating wound healing [31].

### *In vitro* degradation and mineralization

**Figure 3:**
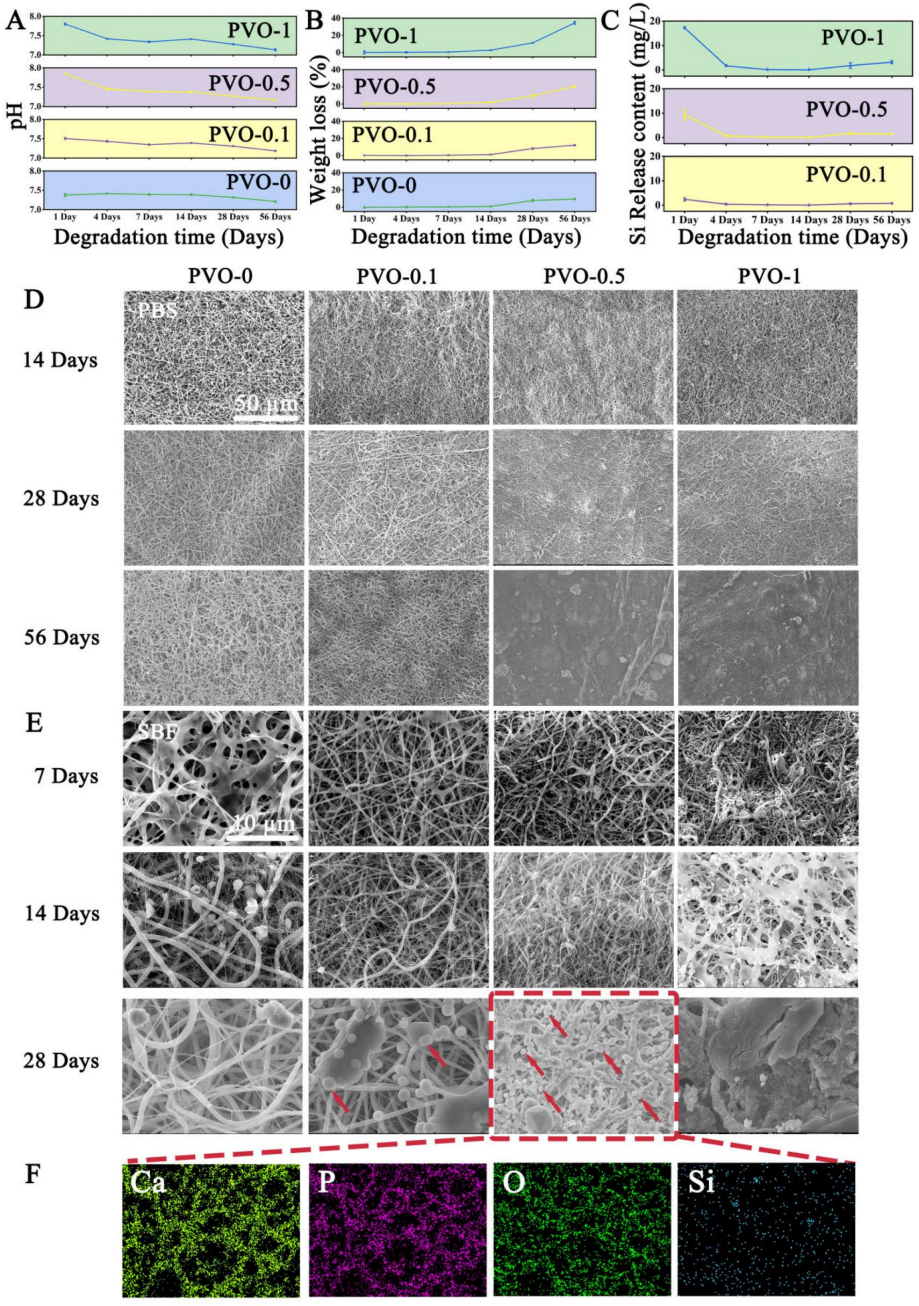
Degradation and mineralization behavior of PVO-X fiber membranes. (**A**) Changes in pH value during degradation; (**B**) Mass loss during degradation; (**C**) Variation of silicon release during degradation; (**D**) Morphological observation of PVO-X fiber membranes degraded in PBS at 14, 28 and 56 days; (**E**) Morphological observation of PVO-X fiber membranes mineralized in SBF at 7, 14 and 28 days (arrows mark hydroxyapatite-like structures); (**F**) EDS analysis of PVO-0.5 fiber membranes after mineralization in SBF for 28 days.

Figure 3A–C showed the pH changes, weight loss rate and silicon release concentration of the PVO-X fiber membrane during its 56-day degradation process. The pH variations across all groups (Figure 3A) exhibited a consistent trend. On day 1, the release of free BG from the fiber surface caused a transient increase in pH [31], with PVO-0.1 showing a pH of 7.5. PVO-0.5 and PVO-1 even reached a pH peak of 7.8. As the degradation time extended, the pH stabilized at approximately 7.4 within 14 days. By day 28, all groups started to lower the pH to 7.3, and it further declined to 7.1 by day 56. The continuous pH reduction resulted from the hydrolytic cleavage of ester bonds in the main chain of PPDO molecules, generating small-molecule carboxylic acid products. The dissociation of carboxylic acids released H^+^ ions, leading to an increase in the hydrogen ion concentration in the solution [30–32]. The pH of all groups remained between 7 and 7.8 during degradation. The combined application of BG and PPDO also mitigated excessive acidity in the wound microenvironment during PPDO degradation [31].

As shown in Figure 3B, the weight loss rate was nearly zero during the first 7 days. However, at day 14, all PVO-X samples began to lose weight, with progressive weight loss increasing. By day 28, the weight loss trend significantly intensified, with PVO-1 and PVO-0.5 exhibiting significantly higher weight loss effects than PVO-0.1 and PVO-0. By day 56, the weight loss reached its peak, with PVO-0.5 achieving a mass loss rate of 20%. The weight loss observed during *in vitro* degradation was primarily attributed to the hydrolytic degradation of PPDO and the dissolution of low-molecular-weight substances [30, 32].

The macroscopic photographs, stress–strain curves and SEM images of PVO-X membranes at different degradation stages are presented in Figure 3D and Supplementary Figures S11–S18, which intuitively illustrate the structural evolution of the materials throughout the degradation process.

On days 1, 4 and 7 of degradation, no significant changes were observed in the macroscopic morphology of the fiber membranes across all groups. Mechanical property tests were conducted at these three time points. The results showed that after 1-day immersion, the overall macrostructure remained intact, but the mechanical properties exhibited a marked decline: the fracture strain of the PVO-0.5 group decreased from 106% to 10%, with the maximum stress remaining around 5 MPa. This performance deterioration primarily stemmed from the altered crystallinity of PPDO due to the 60°C drying treatment, rather than being solely attributable to material degradation [41]. At 14 days of degradation, only mild buckling deformation was observed on the fiber membrane surfaces across all groups, with the overall fiber network structure remaining intact. By day 28, signs of membrane collapse began to appear, accompanied by localized fiber breakage, indicating that the degradation process had triggered significant structural changes. By day 56, all groups exhibited marked morphological damage: PVO-0 and PVO-0.1 groups retained their original fiber morphology with only minor localized melt adhesion, while PVO-0.5 and PVO-1 groups showed extensive melt fusion, making original fiber contours indistinguishable. The fiber networks underwent severe deformation and multiple fractures, forming a continuous sheet-like structure. These findings aligned with observations of pH changes and weight loss trends, with the differences in weight loss rate and morphology between PVO-X groups stemming from variations in fiber diameter caused by the electrospinning process. Fiber diameters of PVO-0 to PVO-1 gradually decreased with increasing BG. The finer fiber diameters and large-pore electrospun structures facilitated water molecule permeation, thereby accelerating the degradation rate.

The silicon release profile in Figure 3C showed that the initial peak in Si^4+^ concentration on day 1 was attributed to the rapid release of unbound BG, which subsequently stabilized between days 4 and 14. On day 28, partial hydrolysis of PVO-X led to gradual release of BG within PPDO fibers. By day 56, further collapse of the PVO-X surface structure released additional BG, achieving sustained release. The average Si^4+^ concentration between days 28 and 56 was 0.6 mg/L for PVO-0.1, 1.5 mg/L for PVO-0.5, and ranged from 1.8 mg/L to 3.2 mg/L for PVO-1. Previous studies have demonstrated that Si^4+^ ions concentrations around 1 mg/L exhibit favorable pro-proliferative effects [31, 34], confirming that PVO-0.5 possesses excellent BG sustained-release capability and may play a positive role in long-term bone repair.

Figure 3E showed abundant hydroxyapatite-like structures observed on the surface of PVO-0.5 fiber membranes during the 28-day *in vitro* SBF experiment. The red arrow indicated the generated hydroxyapatite-like structures. EDS analysis of these spheres (Figure 3F) identified calcium and phosphorus as the primary elements, confirming their composition as calcium phosphate salts. This biomineralization process was triggered by the BG in the fiber membrane, which dissolved the fiber network and released silicate ions (SiO_4_^4−^) and calcium ions (Ca^2+^). The resulting surface silicoxy groups facilitated nucleation, and when the calcium–phosphorus ion product exceeded the solubility product, amorphous calcium phosphate began to deposit and gradually crystallized into osteoid phosphate [42–45]. The formation of this osteoid phosphate directly demonstrated *in vitro* bioactivity, establishing it as the internationally recognized standard for evaluating the biocompatibility of bone repair materials.

*In vitro* cell experiments, degradation silicon release results, and *in vitro* mineralization results all demonstrated that the prepared PVO-0.5 composite fiber membrane exhibits excellent biological activity, indicating promising bone integration potential. The apatite-like layer formed on the material surface first enables direct chemical bonding with newly formed bone tissue, achieving robust bone integration. The porous structure of the fiber membrane provides a vast specific surface area and space for ion exchange and mineralization product deposition, thereby facilitating the entire bone healing process.

### *In vitro* osteogenic differentiation capacity

**Figure 4:**
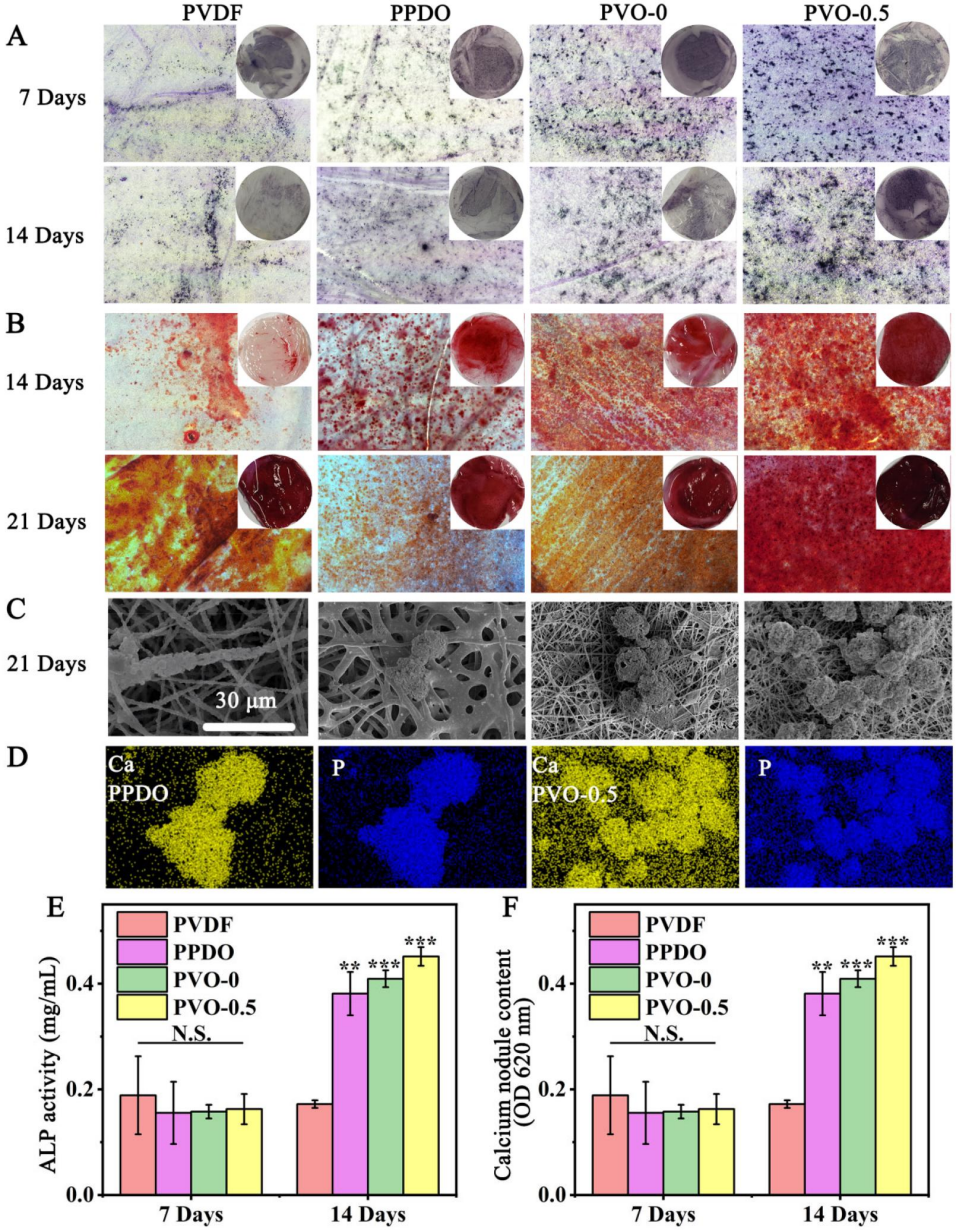
Osteogenic differentiation capacity of PVO-X fibrous membranes. (**A**) ALP staining of cells cultured on PVO-X fibrous membranes. (**B**) Calcium nodule staining. (**C**) SEM images of calcium nodules after 21 days of culture. (**D**) EDS spectra of calcium nodules. (**E**) Quantitative analysis of ALP activity. (**F**) Quantitative analysis of calcium nodule deposition.

Studies have demonstrated that ALP serves as a critical biochemical marker of osteoblast activity during the initial phase of osteogenesis [37, 46, 47]. Consequently, ALP expression is primarily assessed through ALP staining and quantitative activity measurement. ALP staining was performed on cells cultured for 7 and 14 days on three types of membranes, as illustrated in Figure 4A. When the culture duration was extended from 7 to 14 days, the blue–purple areas observed in all groups increased significantly. Notably, the purple region on the PVO-0.5 membrane was more pronounced compared to pure PPDO or PVDF membranes. This trend was also observed in the quantitative monitoring of ALP activity (Figure 4E). After 7 days of culture, the ALP activity expressed by cells on PPDO, PVDF, PVO-0 and PVO-0.5 showed no significant difference, all around 0.15 mg/mL. It was not until the culture time was extended to 14 days that differences began to emerge. PVDF, due to its strong hydrophobic surface, hindered cell adhesion and proliferation, resulting in persistently low ALP activity values at 14 days. In contrast, PVO-0.5 exhibited a more hydrophilic surface, facilitating cell adhesion. Under electrical stimulation, PVO-0 demonstrated stronger ALP activity compared to PPDO. Notably, under the combined effect of electrical stimulation and ionic concentration, the PVO-0.5 group achieved an ALP activity of 0.45 mg/mL, twice that of the PVDF group and slightly higher than the PVO-0 group. This confirms that the addition of BG is effective for osteogenic differentiation, indicating that electrical stimulation and BG loading could jointly enhance the expression of ALP activity and elevate early-stage ALP activity during the osteogenic differentiation process.

During the late osteogenic phase, calcium deposition becomes a key indicator, which can be detected through ARS staining and activity assays [37, 46, 47]. To this end, BMSCs cultured on fibrous membranes for 14 and 21 days were subjected to ARS staining, and the content of deposited calcium nodules was quantitatively assessed by dissolving the stained dye. The morphology of calcium nodules deposited on the cell surface was examined via SEM. Figure 4B showed the ARS staining images of BMSCs cultured on fibrous membranes for 14 and 21 days, revealing a dense and bright red appearance (calcium nodules and ARS chelated products) in all four groups, with PVO-0.5 exhibiting the highest density and brightness. This indicates that the loading of BG promotes the deposition of calcium nodules, consistent with the *in vitro* mineralization results. Quantitative testing of calcium nodules also yielded similar conclusions (Figure 4F). During the culture period from day 14 to day 21, calcium nodule content was expressed as the absorbance of Alizarin Red S staining at 620 nm (OD 620 nm). The absorbance of the PVDF group increased from 0.1–0.5, which was slightly higher than that of the PPDO group (0.4), indicating that electrical stimulation effectively promotes osteogenic differentiation. In the PVO-0.5 group, the absorbance increased from 0.8–1.6 (doubled). Under the synergistic effect of electrical stimulation and ionic concentration, the calcium nodule absorbance in the PVO-0.5 group reached 1.6, compared to only 0.5 in the pure PVDF group and 0.4 in the pure PPDO group. These results demonstrate that the combination of electrical stimulation and BG loading jointly promotes calcium nodule deposition and effectively facilitates the subsequent mineralization process of BMSCs.

After 21 days of *in vitro* culture, as shown in Figure 4C and D, obvious mineralized nodules were observed on the surface of composite fibrous membranes seeded with BMSCs. The deposition process of hydroxyapatite in this study conforms to the multi-stage biomineralization nucleation theory proposed by Reznikov following the classical mineralization pathway of organic matrix guidance → ion enrichment → mineral nucleation → crystal growth and maturation [44].

The bioactive ions continuously released from the scaffold BG component locally accumulate at the type I collagen defect sites secreted by cells, further inducing heterogeneous nucleation and ultimately forming osteoid hydroxyapatite with uniform grain size and crystallinity. Combined with Abalymov’s findings on ion-regulated biomineralization and interfacial nucleation of composite scaffolds, silicon ions released from BG can stabilize amorphous calcium phosphate intermediates and delay their rapid phase transformation into hydroxyapatite, facilitating the formation of nanoscale mineral structures similar to natural bone. Moderately elevated Ca^2+^ concentration in the microenvironment can not only upregulate the expression of osteogenesis-related genes, but also induce the directional deposition of minerals on the fiber scaffold surface through the interfacial charge effect [45].

Notably, the PVO-0.5 group exhibited the best mineral deposition and osteogenic differentiation after 21 days of culture, which was attributed to the synergistic regulation of an appropriate ion concentration window, collagen matrix guidance and scaffold interfacial nucleation, consistent with the inherent law of ion-mediated biomineralization. Overall, the osteogenic mineralization of BMSCs is a result of multi-factor coupling. Type I collagen secreted by cells acts as an organic mineralization template, BG ions provide chemical driving force for mineralization, electrospun fibers construct nucleation interfaces, and PVDF piezoelectric signals further exert physical regulation, jointly achieving a multi-stage synergistic biomimetic biomineralization process.

### *In vivo* cranial bone healing capacity

To evaluate the regenerative effect of PVO-X fibrous membrane in bone defect repair, a critical size rat skull bone defect model was established. After 4 weeks and 8 weeks of implantation, HE staining and Masson staining were performed, and statistical analyses were conducted on new bone mass, trabecular bone maturity, neovascularization, and bone formation to systematically assess its *in vivo* osteogenic healing capacity. Specific scoring criteria are detailed in Supplementary Table S2.

**Figure 5:**
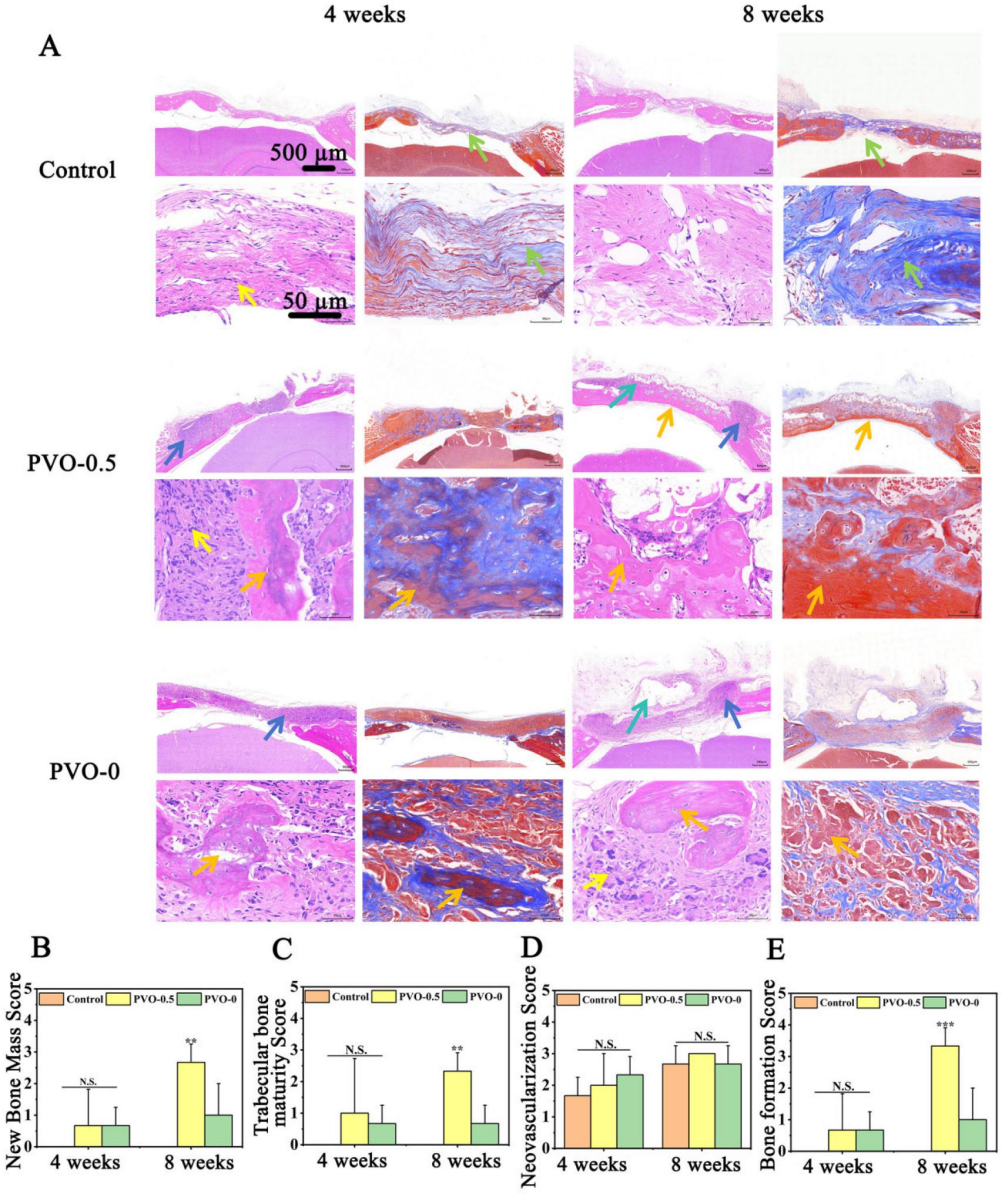
*In vivo* osteogenesis of PVO-X fibrous membranes. (**A**) Histological staining images of control, PVO-0.5, and PVO-0 HE groups (HE and Masson staining). (**B**) Quantitative analysis of new bone mass. (**C**) Quantitative analysis of trabecular bone maturity. (**D**) Quantitative analysis of neovascularization. (**E**) Quantitative analysis of bone formation. (green arrow: fibrous tissue; orange arrow: new bone tissue; yellow arrow: macrophages; blue arrow: granuloma; cyan arrow: fibrous capsular cavity).

At 4 weeks postoperatively, as shown in Figure 5, the defect area in the control group was dominated by fibrous connective tissue (green arrows), accompanied by mild macrophage infiltration (yellow arrows). The repair process stagnated at the fibrous healing stage, and the new bone mass score was 0.00, indicating a lack of effective bone regeneration. In contrast, the PVO-0.5 and PVO-0 groups showed more active early healing responses with obvious neovascularization. A neatly arranged osteoblast layer was observed at the defect edge, indicating the initiation of bone repair. The neovascularization scores were 2.00 for PVO-0.5 and 2.33 for PVO-0, both higher than 1.67 for the control group. Notably, the PVO-0.5 group exhibited small amounts of new bone at the material–bone interface (orange arrows). Although the trabecular structure was sparse, osteogenic activity had been activated. The PVO-0 group also showed new bone formation, but its trabecular maturity and bone formation scores were not significantly different from those of the PVO-0.5 group, suggesting similar osteogenic efficiency at the initial repair stage. At this stage, the fibrous membrane experienced limited degradation, and electrical stimulation served as the primary mechanism. During early bone repair, electrical stimulation generated surface charges that promote the assembly of charged macromolecules via electrostatic interactions and stimulate the migration, proliferation and differentiation of osteoblasts, osteoclasts, and their precursor cells [20, 28, 48, 49].

At 8 weeks postoperatively, as shown in Figure 5, the differences in repair outcomes among groups became highly evident. In the control group, the defect was still filled with fibrous tissue (green arrows) with no new bone formation (orange arrows), confirming that critical-sized defects cannot achieve bony healing under natural conditions. The PVO-0 group showed limited repair efficiency, accompanied by persistent granulomas and fibrous cavities (blue arrows; cyan arrows), with only sparse and discontinuous new bone observed. Quantitative scoring showed new bone mass of 1.00, trabecular maturity of 0.67, and bone formation of 1.00, indicating weak and unstable osteogenic capacity. In contrast, the PVO-0.5 group exhibited significantly better osteogenic performance. Below the space created by material degradation, large areas of continuous and mature new bone with clear lacunae were observed, indicating the formation of a highly mineralized and biologically active bone matrix. The PVO-0.5 group achieved a new bone mass of 2.67, trabecular maturity of 2.33, and bone formation of 3.33, all of which were higher than those of the PVO-0 group. Meanwhile, its neovascularization score remained at 3.00, providing stable nutritional supply for active bone formation. The sustained release of bioactive ions from BG at 8 weeks synergized with the piezoelectric stimulation of PVDF, jointly promoting collagen crosslinking, hydroxyapatite deposition, and osteogenic signal activation, thereby significantly accelerating bone maturation and repair [10, 35, 44, 45].

In conclusion, the histological results demonstrated that the PVO-0.5 composite fiber membrane mediates staged regulation during bone defect repair. At the early stage (4 weeks), piezoelectric stimulation plays a leading role in initiating cell recruitment and early osteogenesis. At the late stage (8 weeks), the sustained release of ions from BG became the key enhancer and synergized with piezoelectric signals to significantly promote bone matrix mineralization and structural bone regeneration. In comparison, PVO-0 without BG cannot provide continuous bioactive stimulation, resulting in limited and unstable osteogenic effects. The synergistic effect of ion signals and electrical signals effectively promotes the synthesis, mineralization and maturation of the bone matrix, thereby achieving high-quality structural repair of critical-sized bone defects.

## Conclusion

In summary, this study developed a piezoelectric sustained-release fiber membrane (PVO-0.5) by integrating PVDF, PPDO, and BG through alternating electrospinning technology. The membrane exhibits both electrical stimulation and controllable ion release capabilities, facilitating regulation of the electrical microenvironment, ionic microenvironment, and ECM microenvironment. *In vitro* experiments demonstrated that PVO-0.5 promoted the proliferation and osteogenic differentiation of BMSCs, with calcium nodule formation observed by day 21. In a rat critical-sized cranial defect model, new bone formation was detected at week 4 post-surgery, with substantial bone repair observed by week 8, suggesting improved bone defect healing and enhanced postoperative bone repair quality at week 8.

However, this study has limitations. The *in vivo* sample size was relatively small (*n* = 3 per group per time point), which may affect the statistical robustness. Future work will include larger animal studies, RT-PCR and Western blot analyses to clarify the molecular pathways, and additional control groups to better separate the contributions of piezoelectric stimulation versus ion release.

Despite these limitations, PVO-0.5 represents a promising scaffold material in the field of bone repair, offering a multifunctional strategy for bone defect healing and showing potential applications in regenerative medicine.

## Supplementary Material

rbag120_Supplementary_Data
