# Supplementary material for: Electrospun PPDO-BG/PVDF fibrous membranes with piezoelectric synergistic bioactive ion controlled release for enhanced bone repair
Source: Regen Biomater. 2026 Jun 9;13:rbag120. doi: 10.1093/rb/rbag120 (PMC13348707; doi:10.1093/rb/rbag120)
Supplement: rbag120_Supplementary_Data [file rbag120_supplementary_data.docx]

Supporting Information

**Electrospun PPDO-BG/PVDF fibrous membranes with piezoelectric synergistic bioactive ion controlled release for enhanced bone repair**

Qiuyu Zeng^a,#^, Junqin Mao^a,#^, Yutong Zhang^a^, Xinyu Zhang^a^, Tao Shen^a^, Heng Zheng^a,^*, Guoyu Lv^a,^[[1]](#footnote-0)^*^

^a^ College of Physics, Sichuan University, Chengdu 610065, China


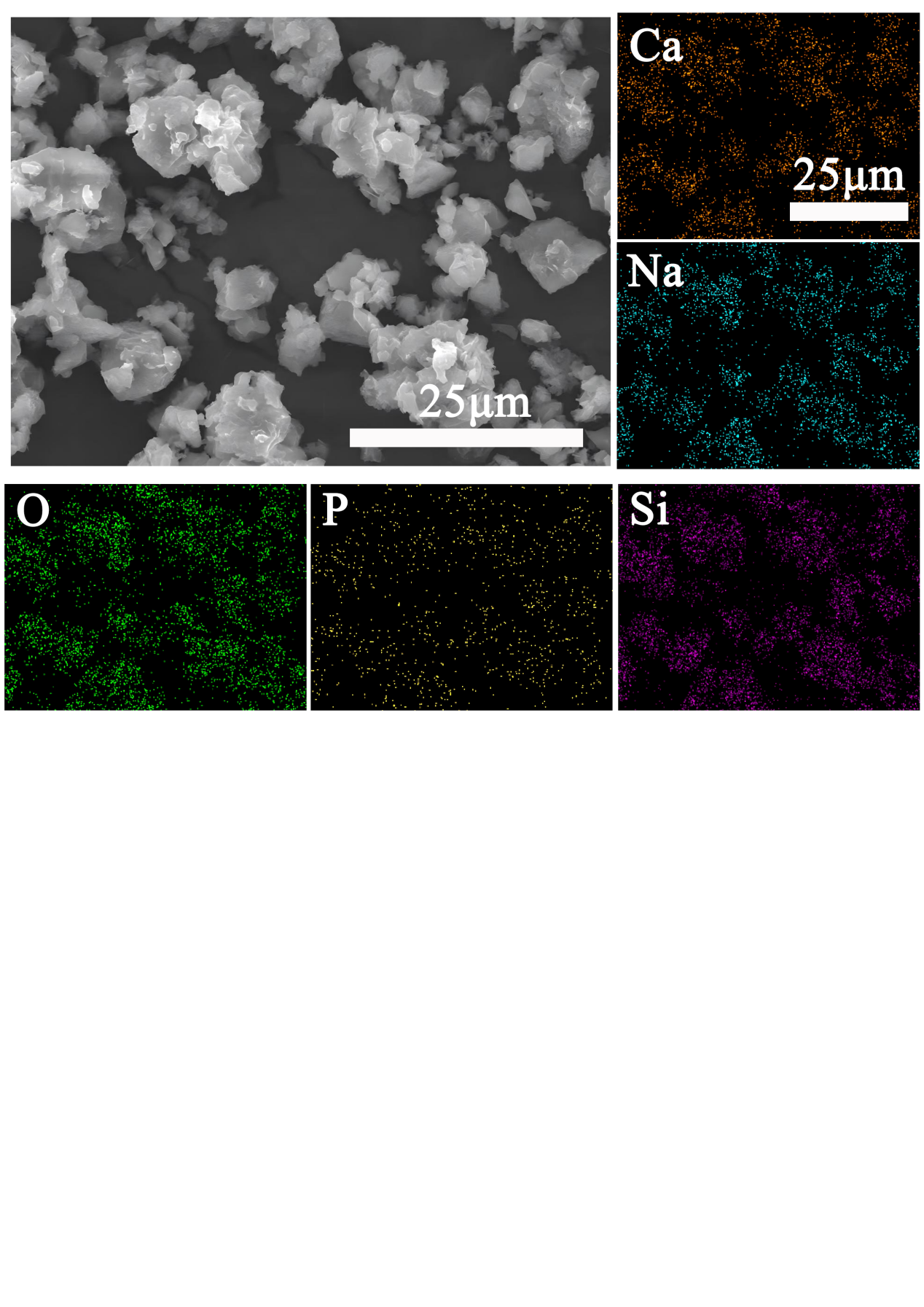


**Figure S1. SEM and EDS images of bioactive glass.**


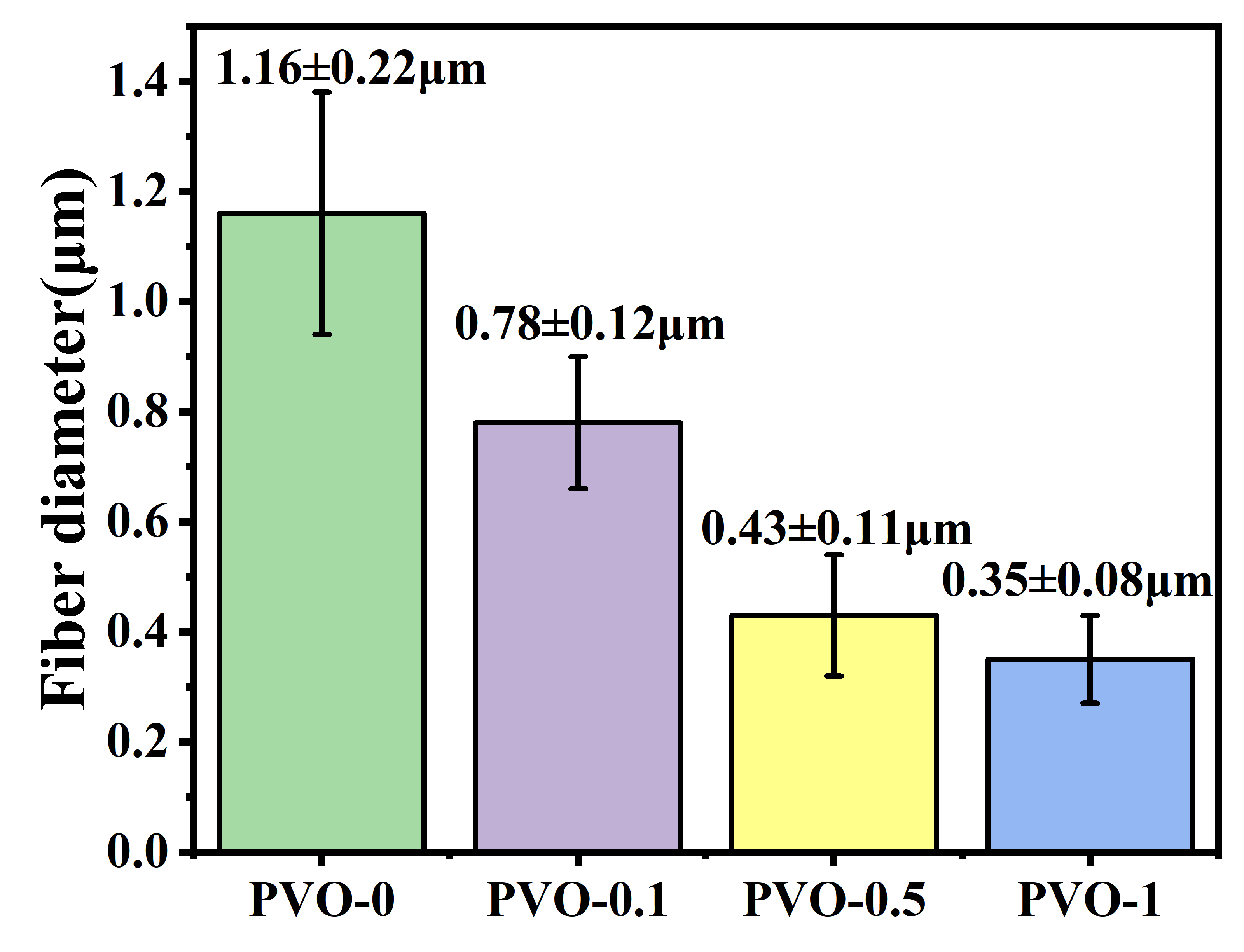


**Figure S2. Diameter statistics of PVO-X fiber membranes.**


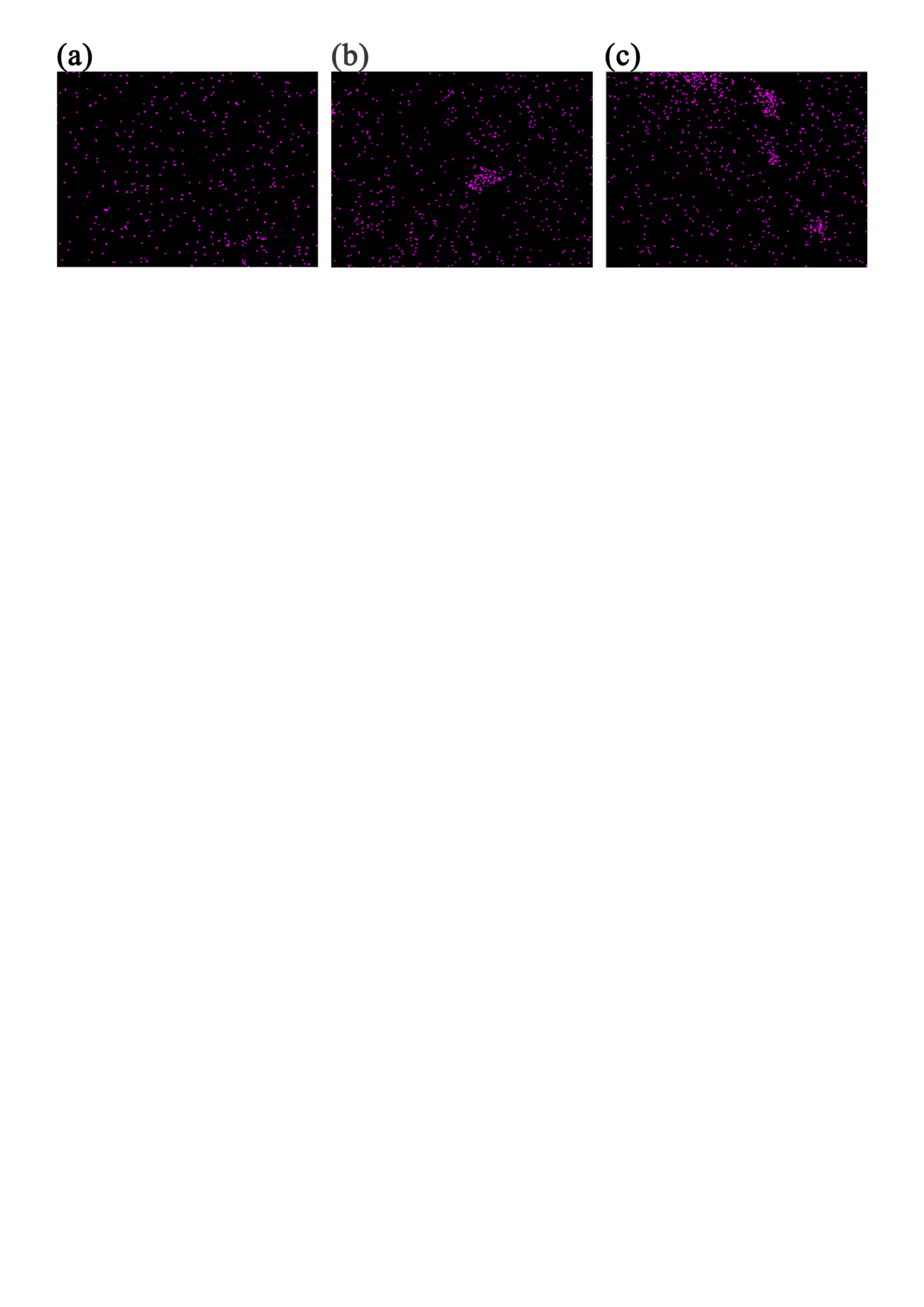


**Figure S3. EDS of Si element in PVO-X fiber membrane.**

**Table S1. Water contact angle of PVO-X fiber membrane**

| **Experimental group** | **Water contact angle** |
| --- | --- |
| **PVO-0** | **(122.57±13.28)°** |
| **PVO-0.1** | **(104.22±3.91)°** |
| **PVO-0.5** | **(72.88±11.58)°** |
| **PVO-1** | **(64.67±3.34)°** |


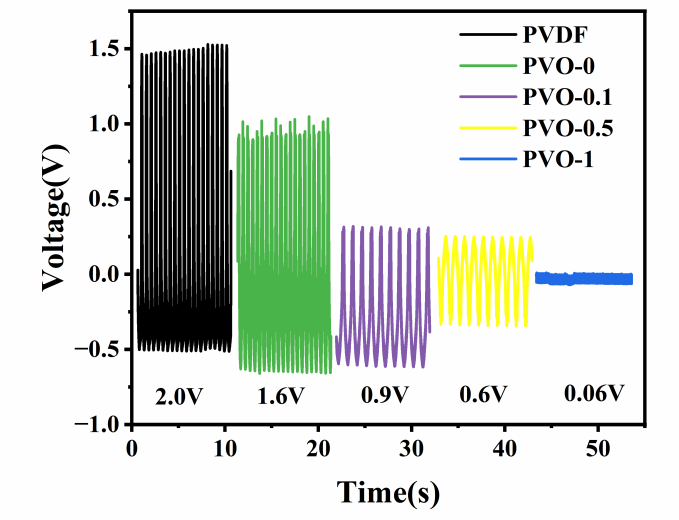


**Figure S4. The specific voltage value of the PVO-X fiber membrane.**


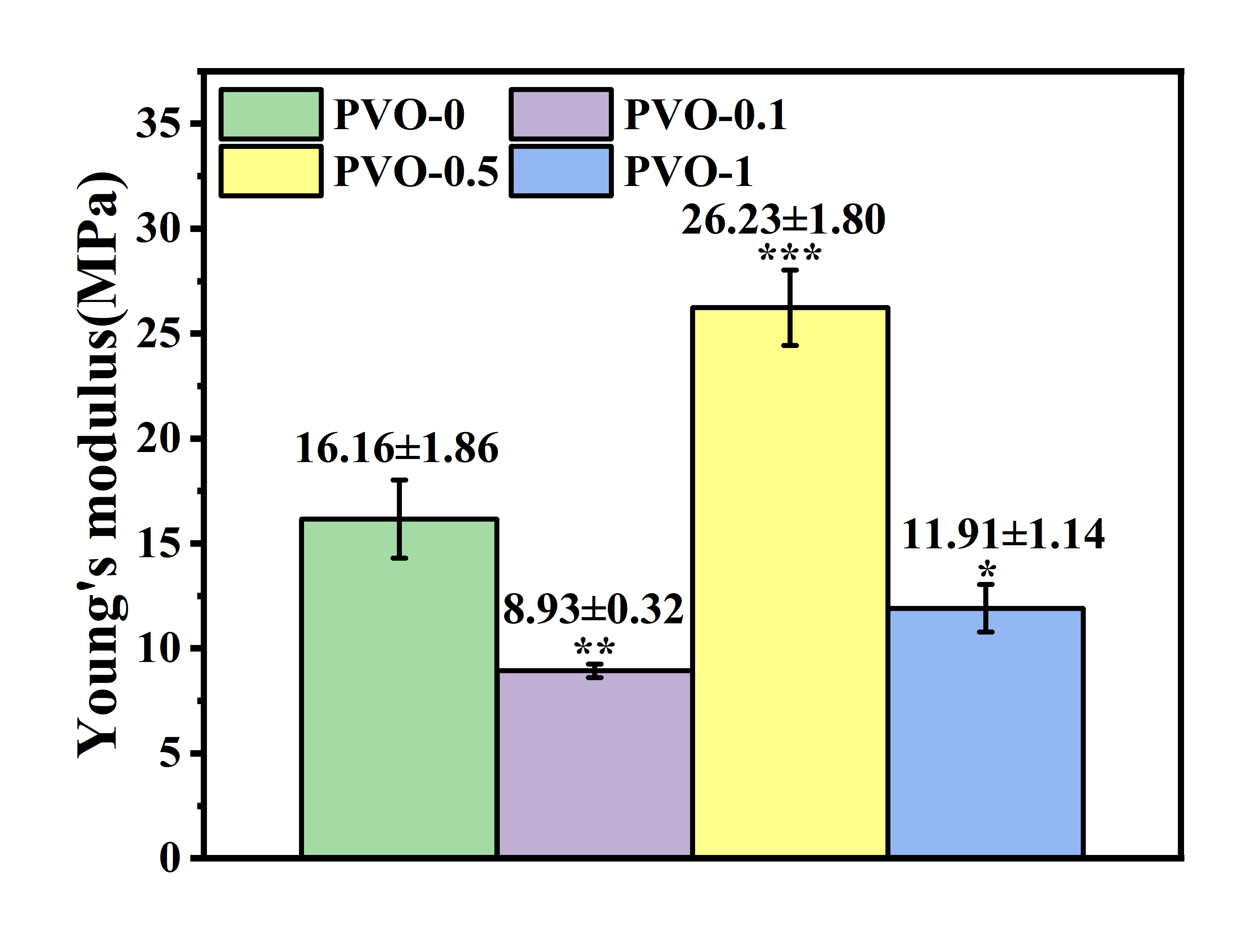


**Figure S5. The Young's modulus of the PVO-X fiber membrane.**


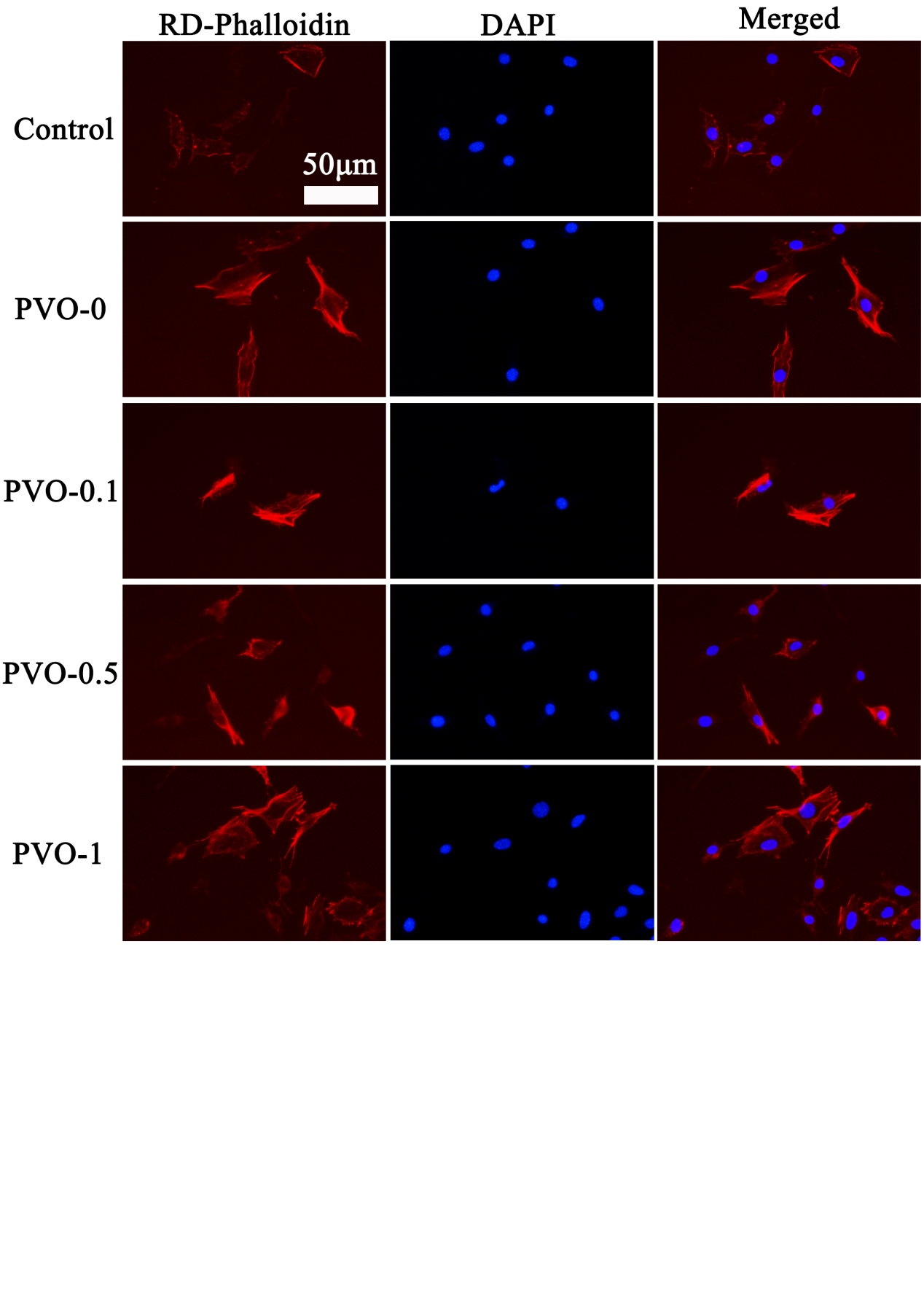


**Figure S6. High-magnification cytoskeleton staining image of a single cell.**


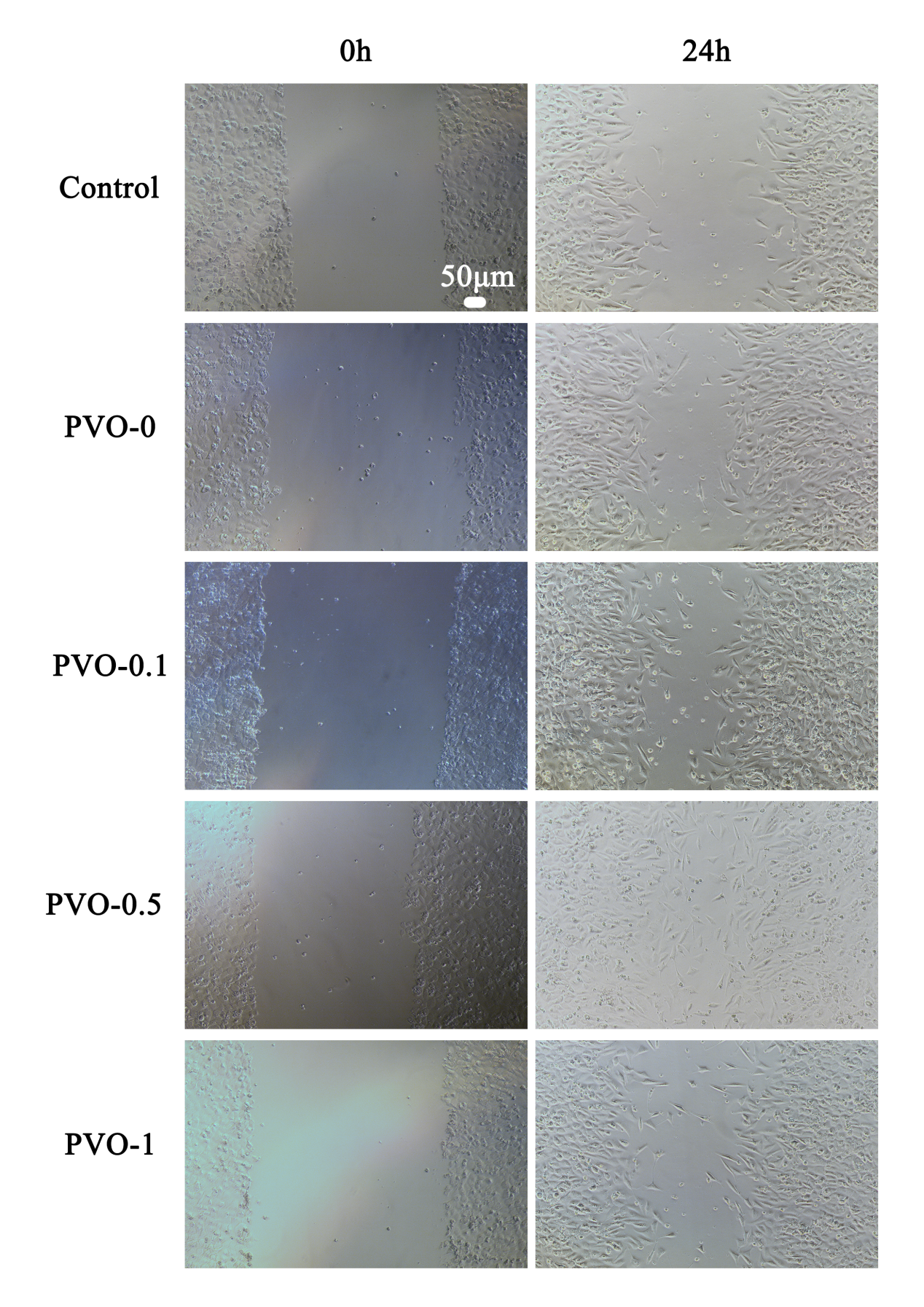


**Figure S7. Microscopic image of the PVO-X fiber membrane.**


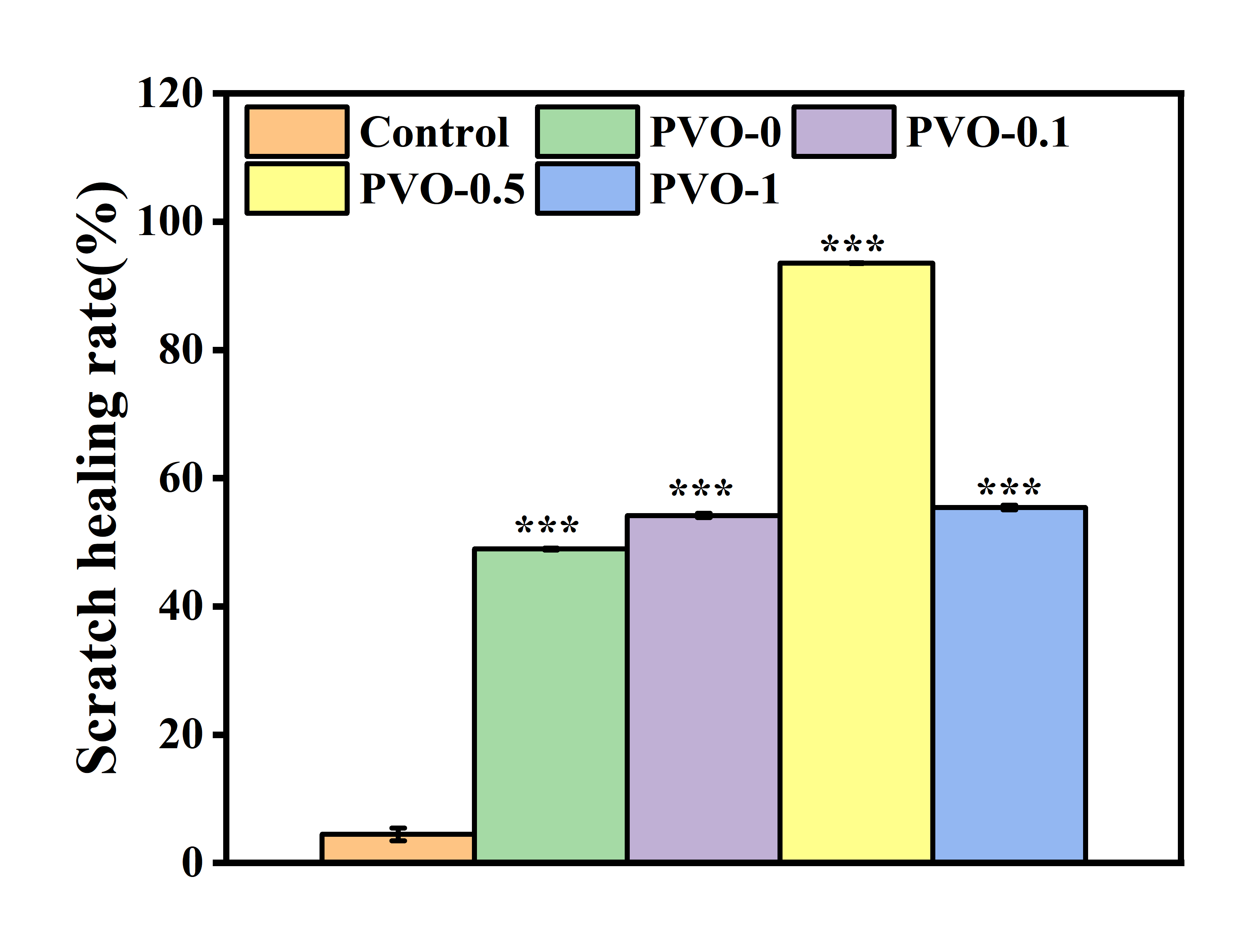


**Figure S8. Scratch healing rate of PVO-X fiber membrane.**


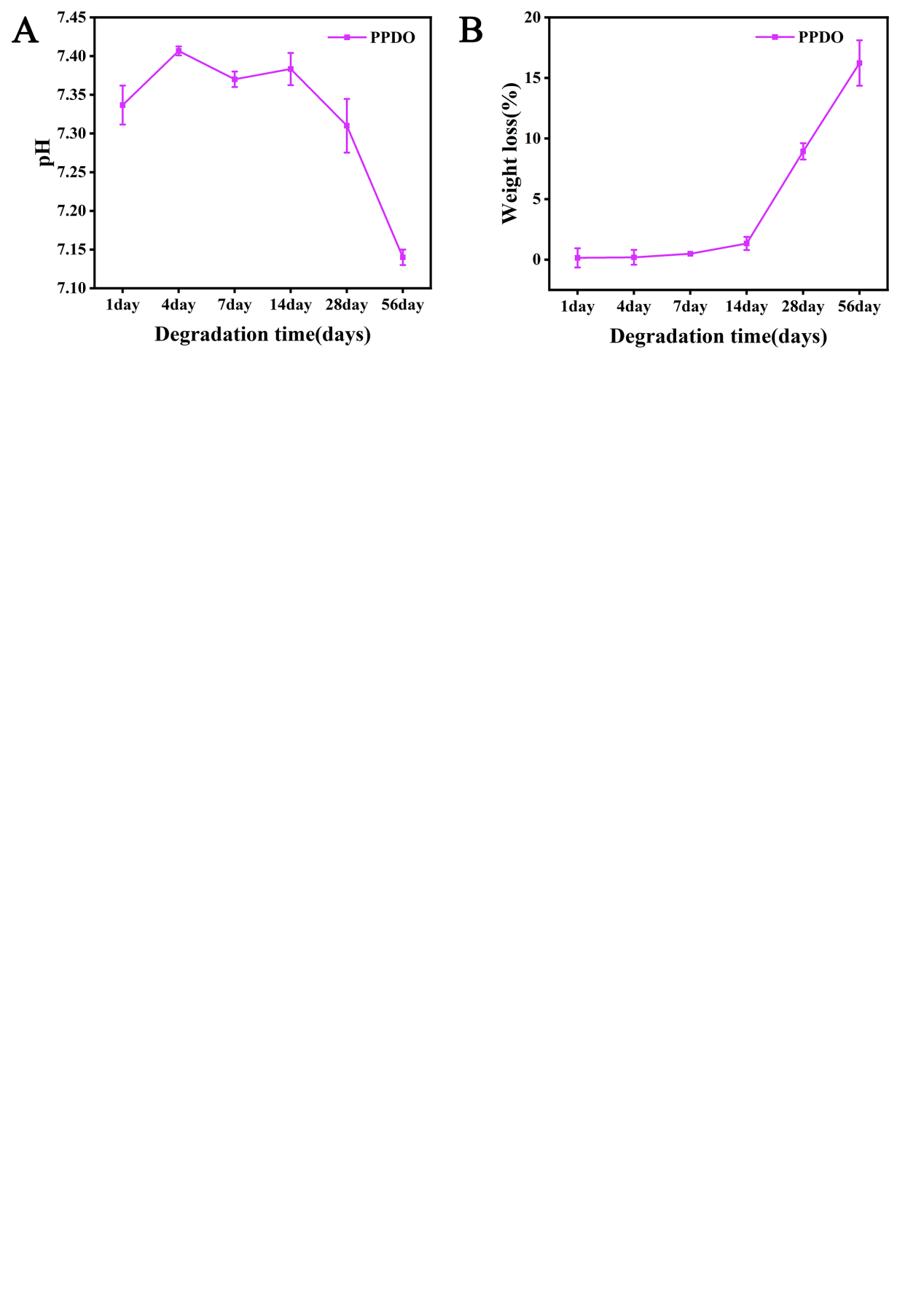


**Figure S9. (A) pH changes during PPDO fiber membrane degradation. (B) Mass loss during PPDO fiber membrane degradation.**


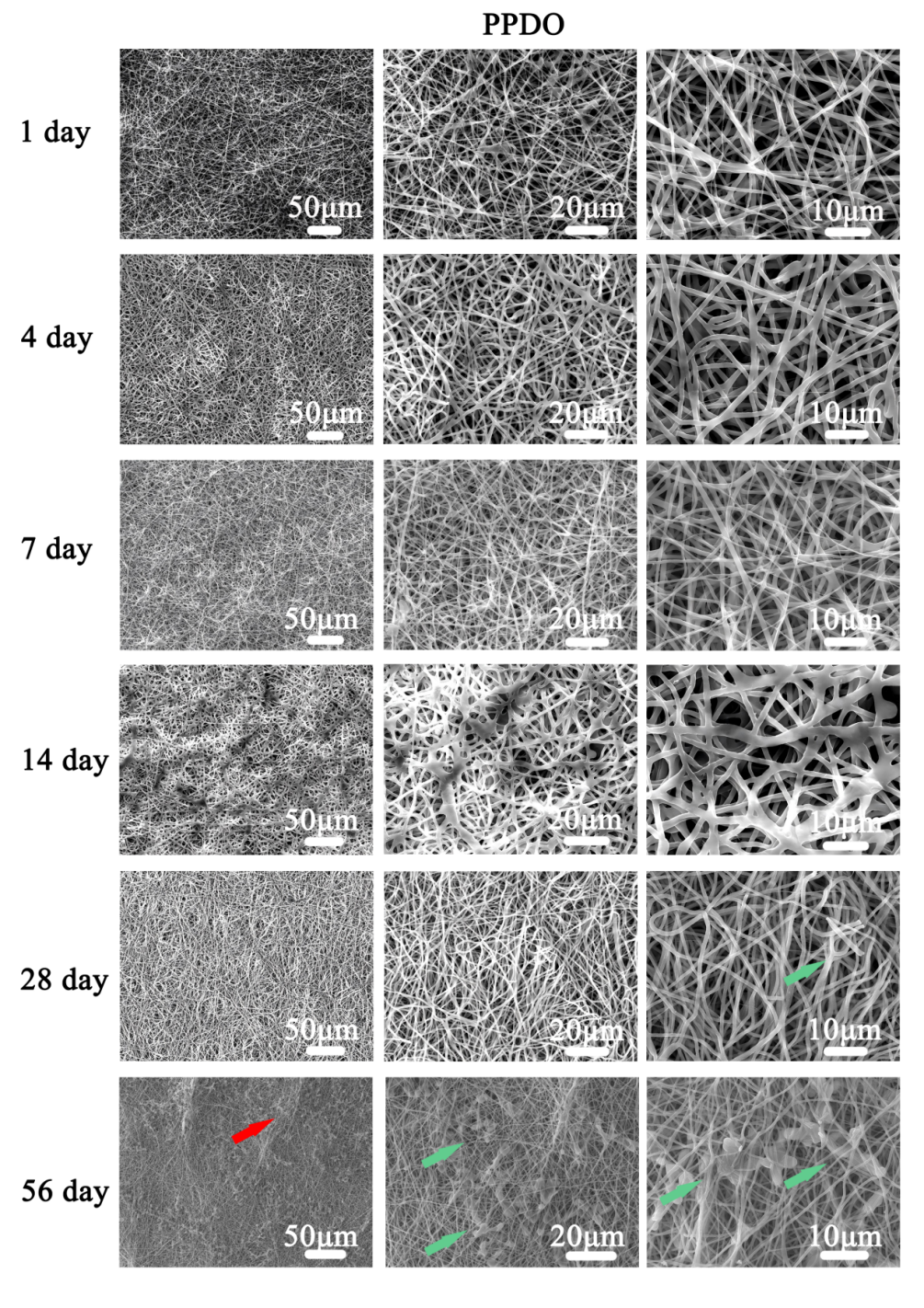


**Figure S10. SEM images of the PPDO fibrous membrane during degradation. (green arrows representing fiber rupture, red arrows representing fiber collapse)**


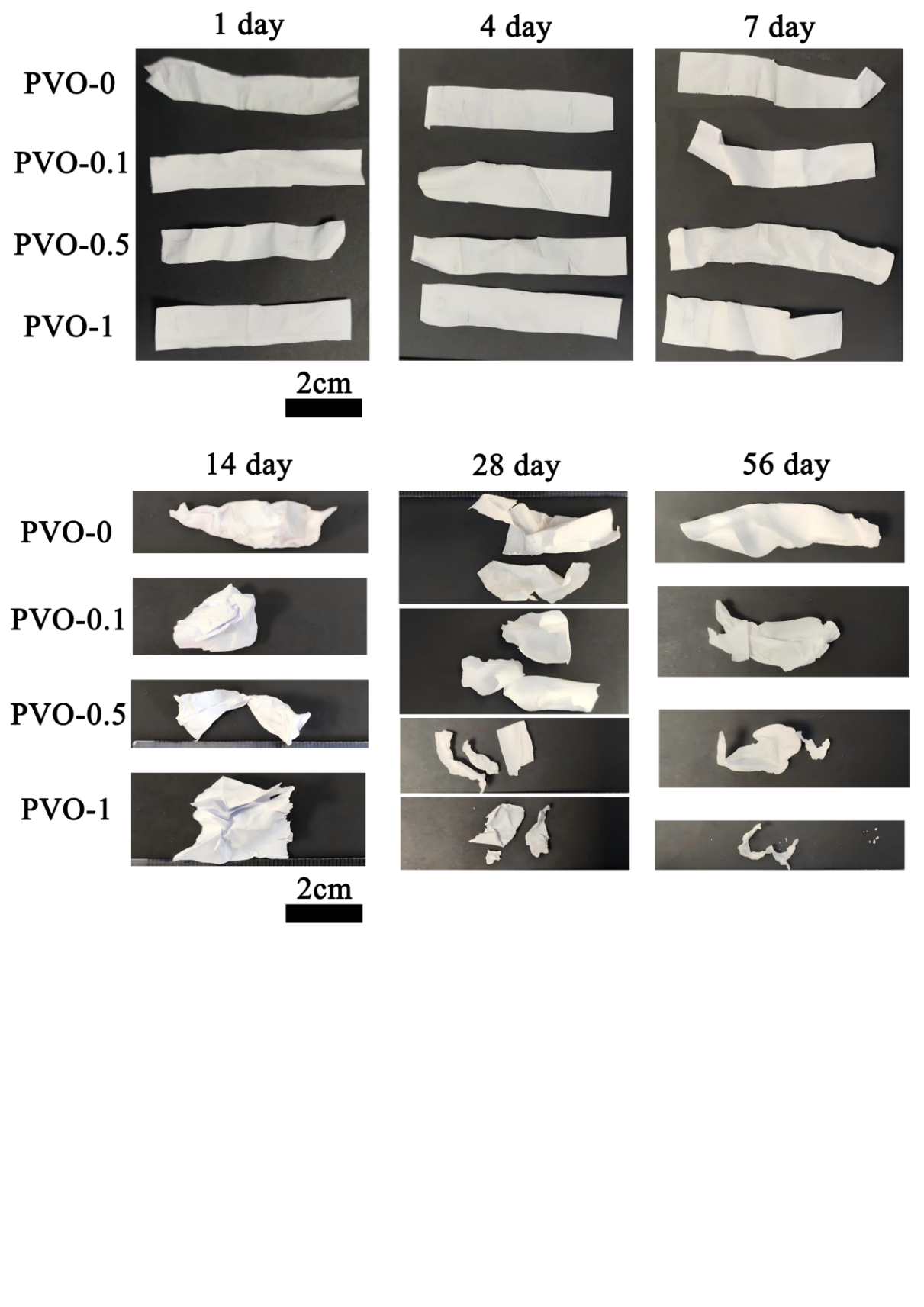


**Figure S11. Macrophotographs of the degradation process of PVO-X fiber membranes.**


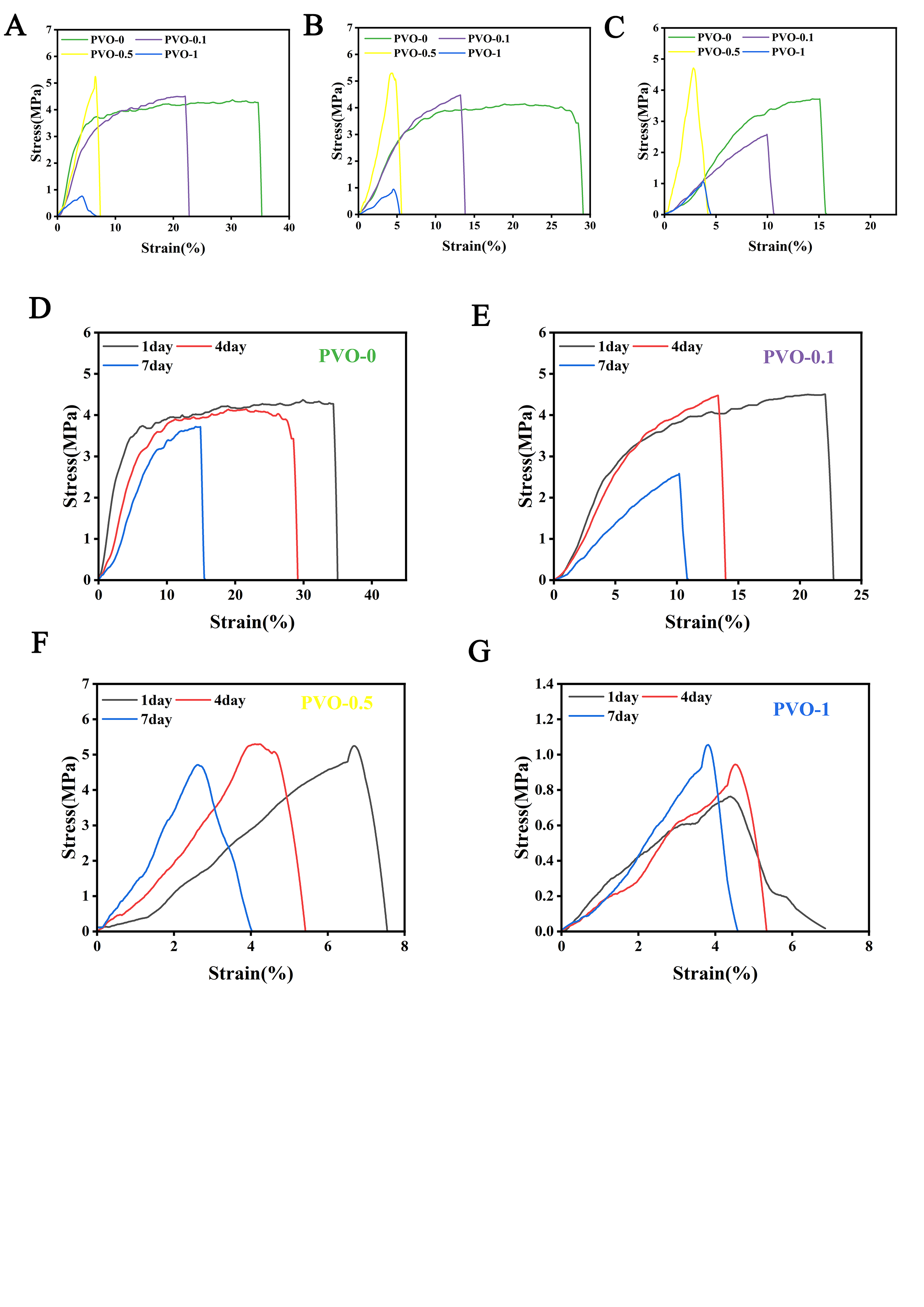


**Figure S12. (A) Mechanical properties of the fiber membranes after 1 day of degradation. (B) Mechanical properties of the fiber membranes after 4 days of degradation. (C) Mechanical properties of the fiber membranes after 7 days of degradation. (D)-(G) Changes in mechanical properties of the fiber membranes at 1, 4, and 7 days after degradation.**

**
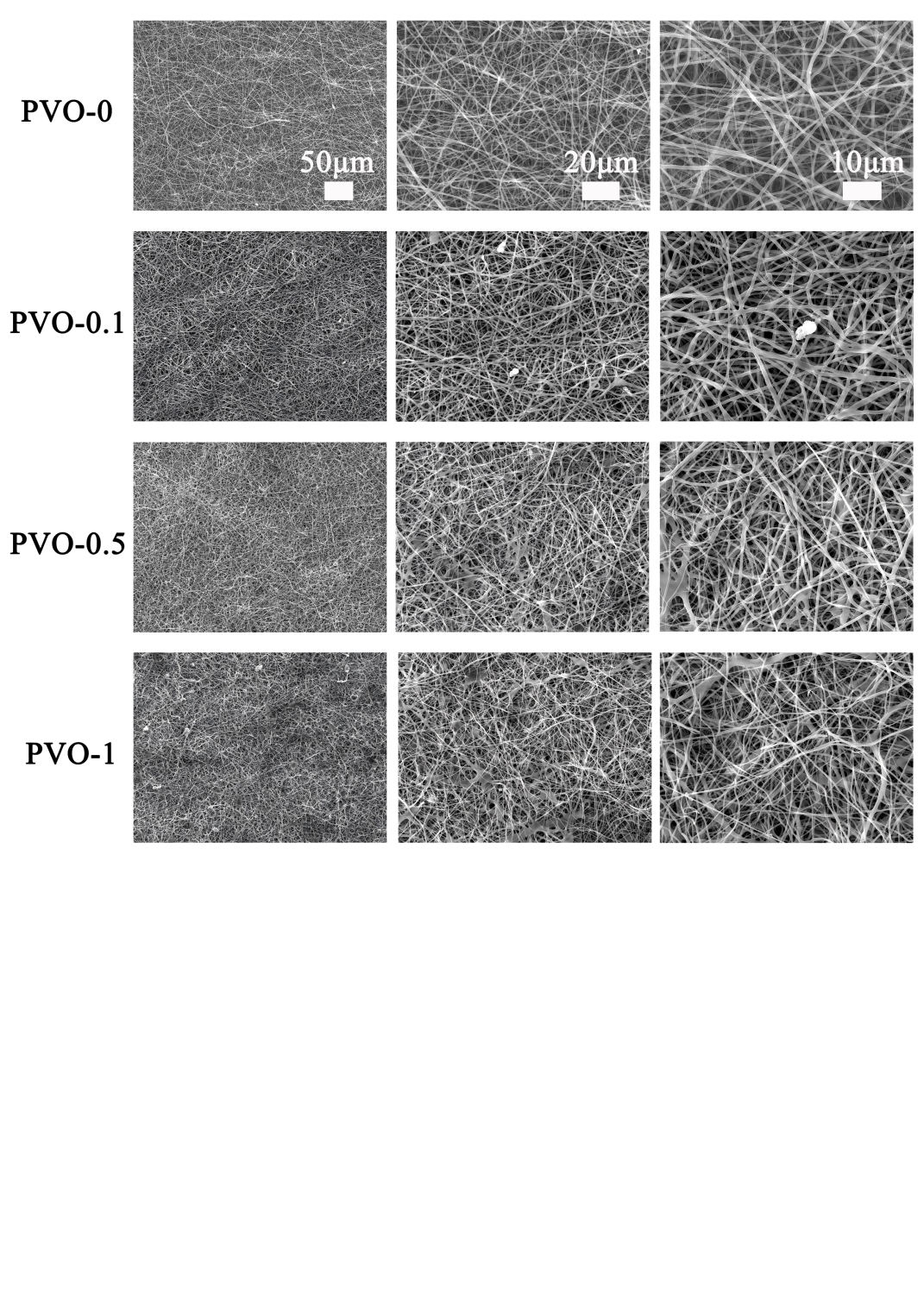
**

**Figure S13. SEM image of PVO-X fiber membrane degradation on day 1.**

**
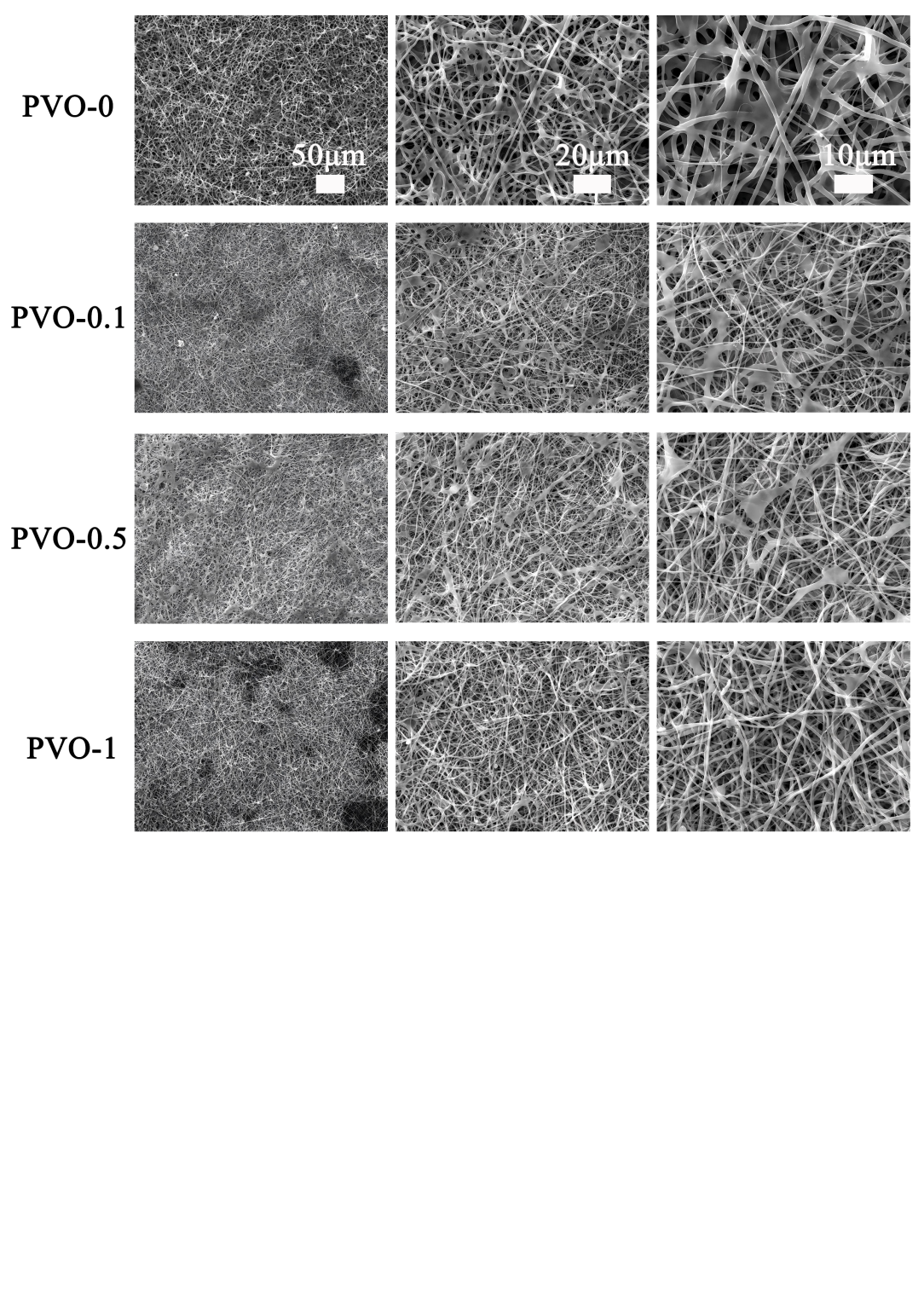
**

**Figure S14. SEM image of PVO-X fiber membrane degradation on day 4.**

**
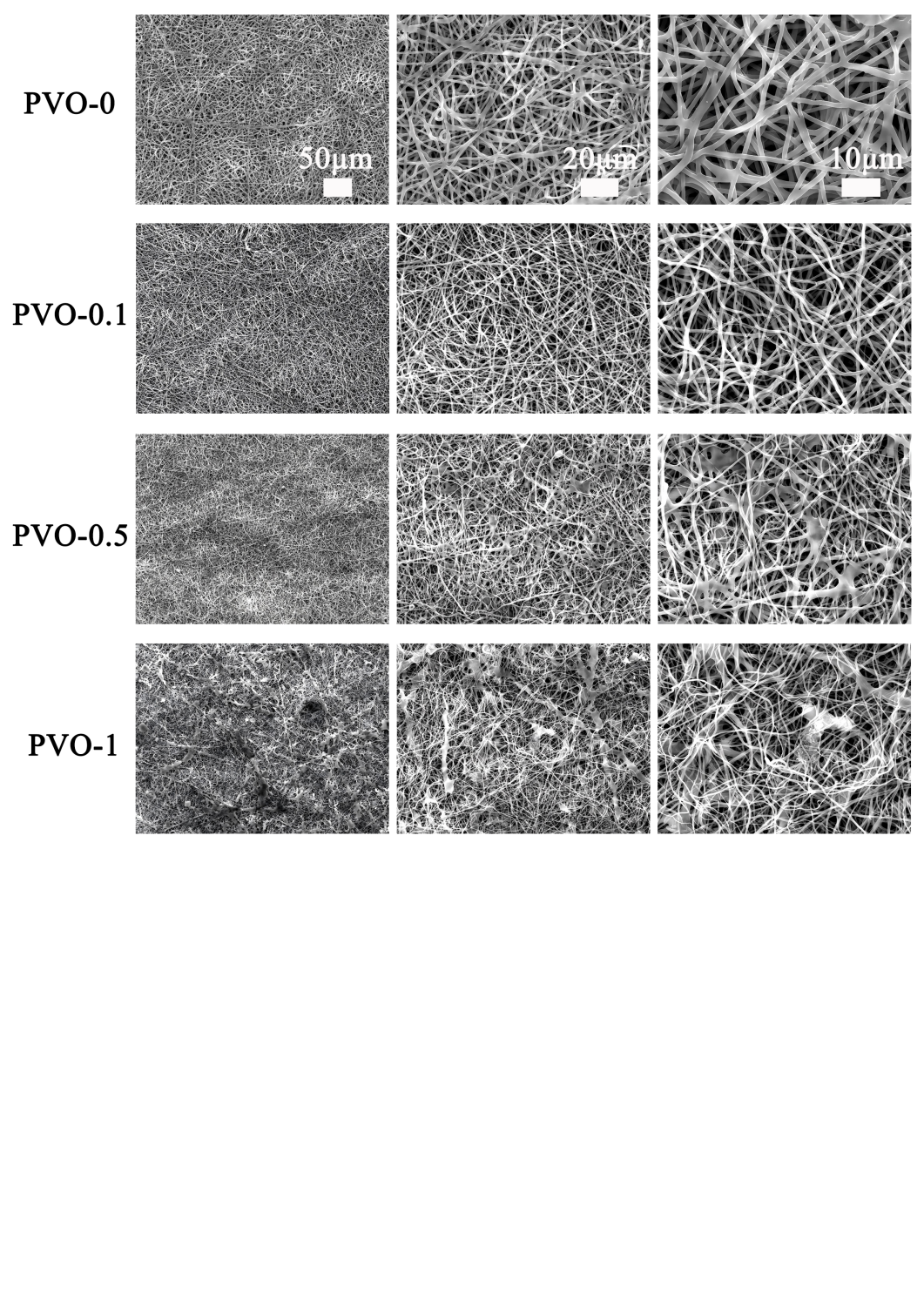
**

**Figure S15. SEM image of PVO-X fiber membrane degradation on day 7.**

**
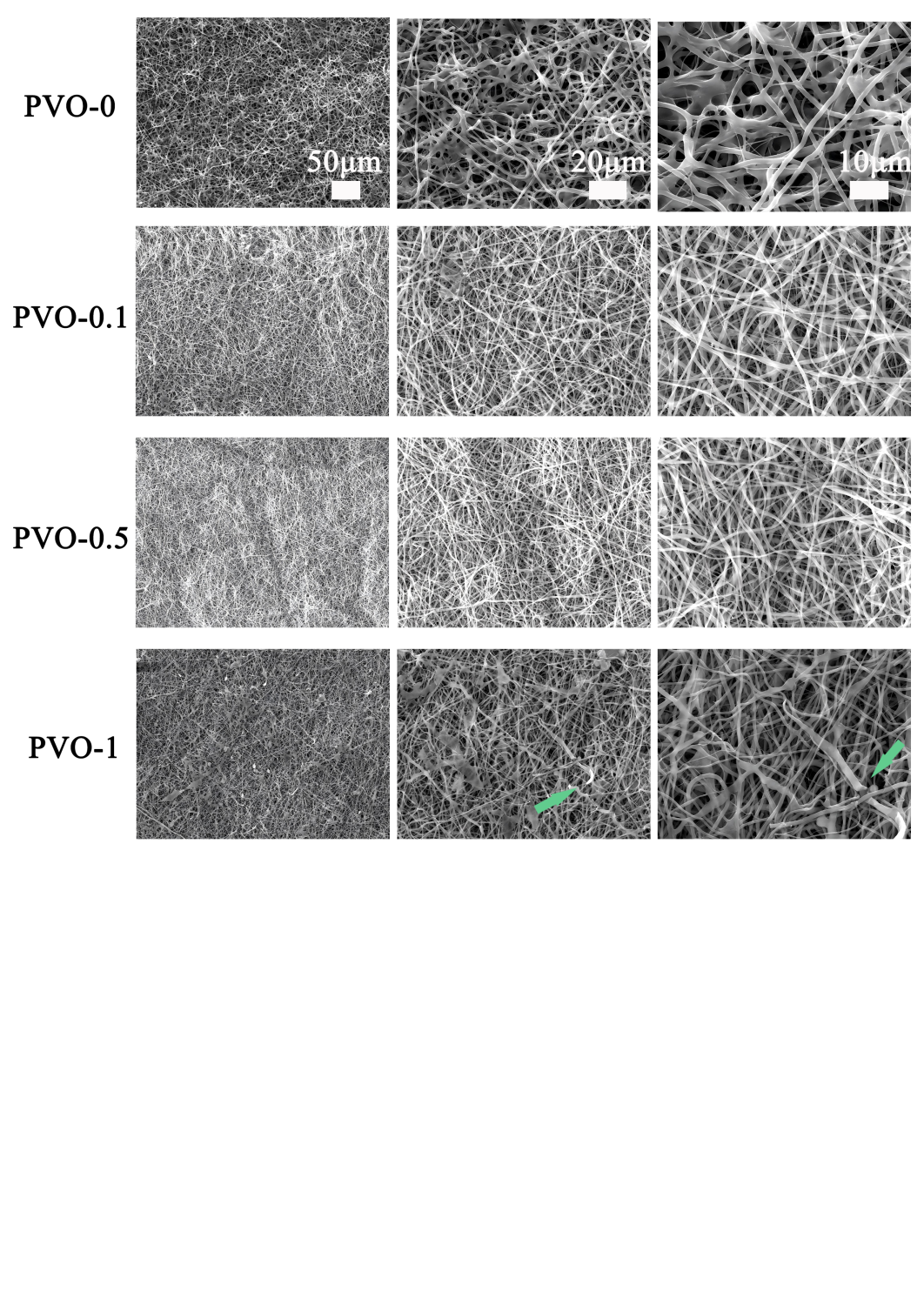
**

**Figure S16. SEM image of PVO-X fiber membrane degradation on day 14. (the green arrow representing fiber rupture)**

**
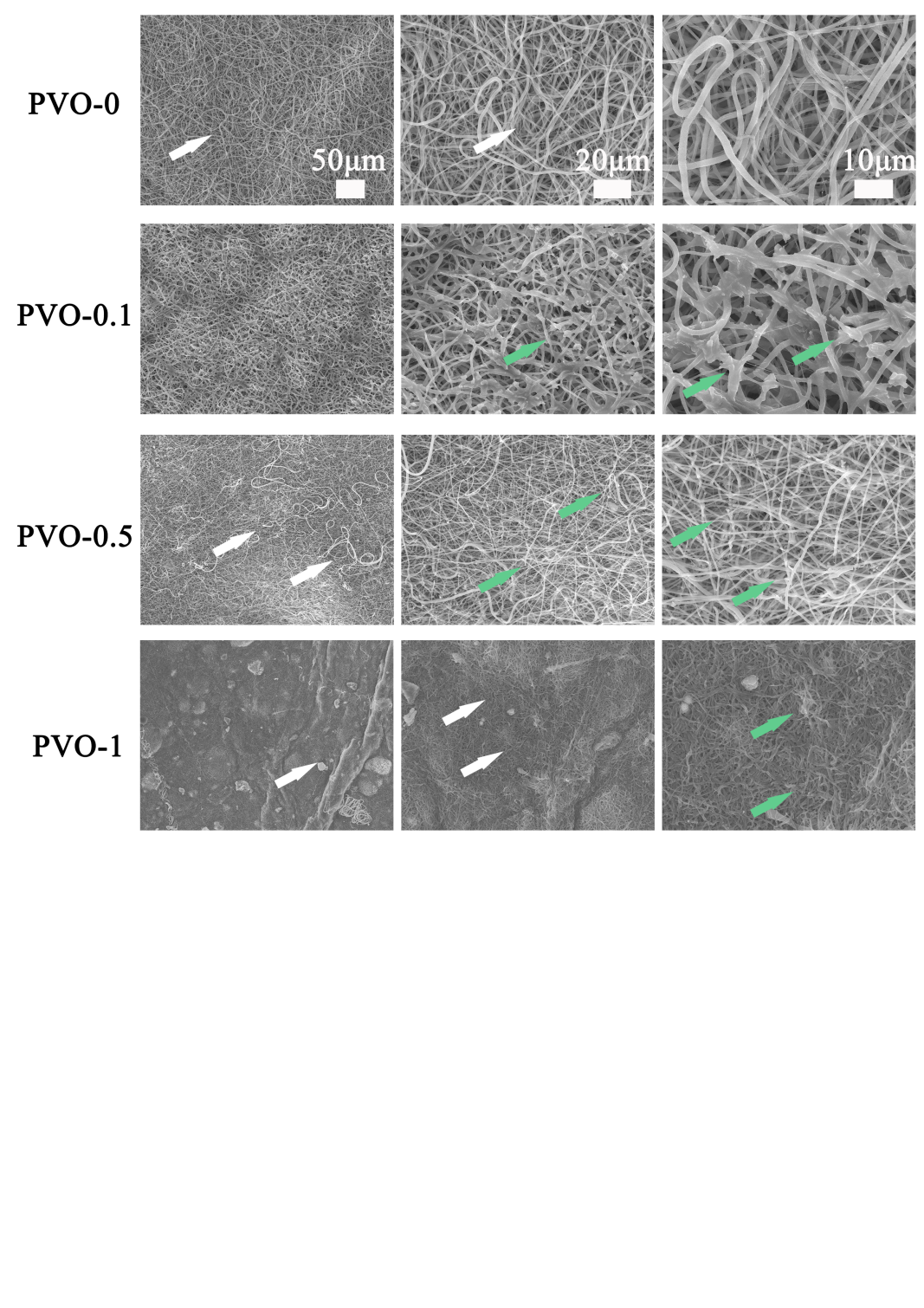
**

**Figure S17. SEM image of PVO-X fiber membrane degradation on day 28. (the green arrow representing fiber rupture, the white arrow representing curling)**

**
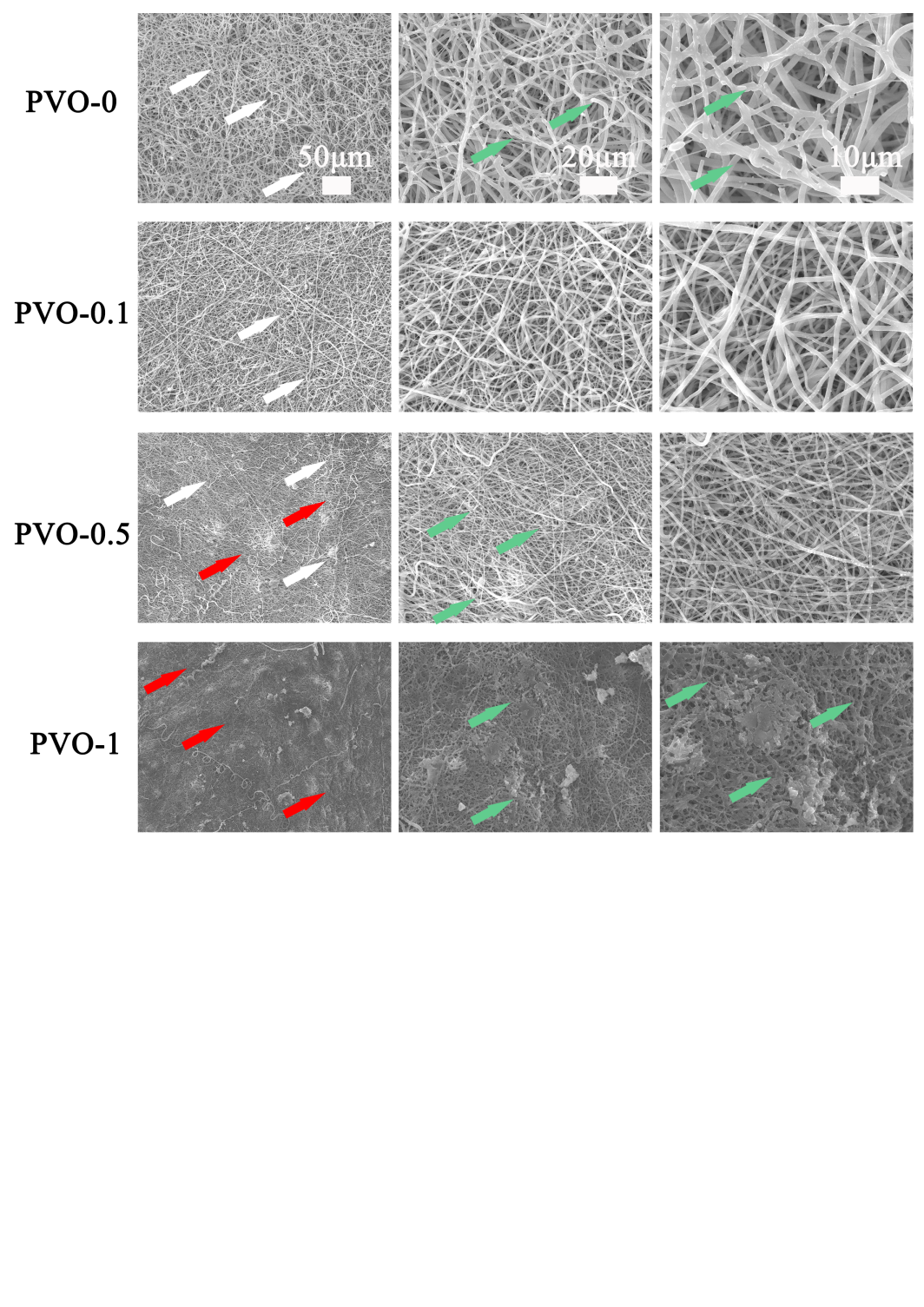
**

**Figure S18. SEM image of PVO-X fiber membrane degradation on day 56. (the green arrow representing fiber rupture, the white arrow representing curling, and the red arrow representing collapse)**


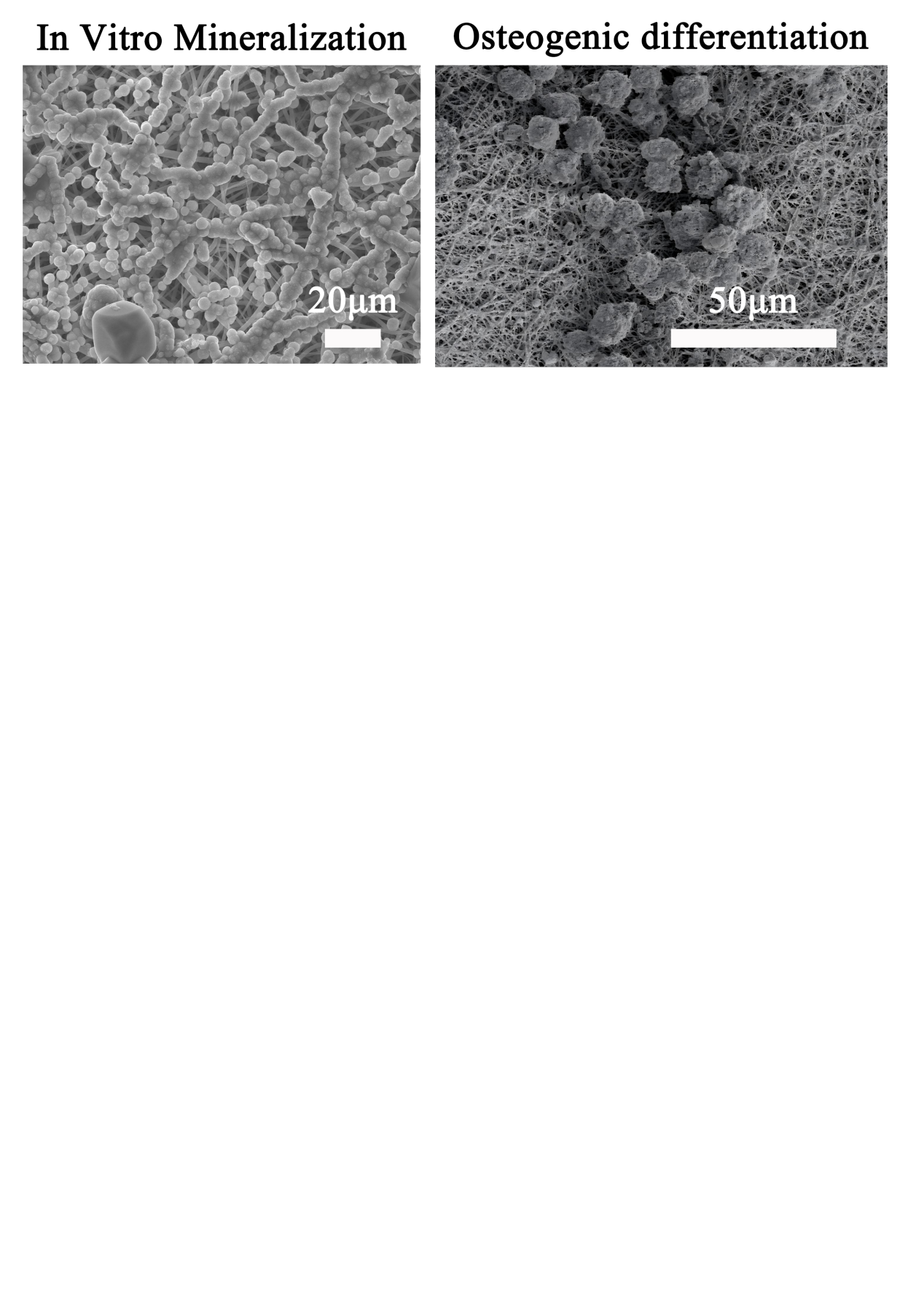


**Figure S19. Mineralization products after 28 days of in vitro SBF experiment (left), and mineralization products after 21 days of BMSCs osteogenic differentiation experiment (right).**

**Table S2. HE scoring results for each group of rats (Mean ± SD)**

| **Time** | **Group** | **New bone mass** | **Trabecular bone maturity** | **Neovascularization** | **Bone formation** |
| --- | --- | --- | --- | --- | --- |
|  |  |  |  |  |  |
| 4w | Control | 0.00±0.00 | 0.00±0.00 | 1.67±0.58 | 0.00±0.00 |
|  | PVO-0.5 | 0.67±1.15 | 1.00±1.73 | 2.00±1.00 | 0.67±1.15 |
|  | PVO-0 | 0.67±0.58 | 0.67±0.58 | 2.33±0.58 | 0.67±0.58 |
| 8w | Control | 0.00±0.00 | 0.00±0.00 | 2.67±0.58 | 0.00±0.00 |
|  | PVO-0.5 | 2.67±0.58 | 2.33±0.58 | 3.00±0.00 | 3.33±0.58 |
|  | PVO-0 | 1.00±1.00 | 0.67±0.58 | 2.67±0.58 | 1.00±1.00 |

1. # These authors contributed equally to the work.

   * Corresponding author. E-mail address: zhengheng@scu.edu.cn (Heng Zheng), lgy929@126.com (Guoyu Lv). [↑](#footnote-ref-0)
